# Integrating Deep Learning and Real-Time Imaging to Visualize In Situ Self-Assembly of Self-Healing Interpenetrating Polymer Networks Formed by Protein and Polysaccharide Fibers

**DOI:** 10.1021/acsami.5c11459

**Published:** 2025-08-05

**Authors:** Gloria Pelayo-Punzano, Rafael Cuesta, José J. Calvino, José M. Domínguez-Vera, Miguel López-Haro, Juan de Vicente, Natividad Gálvez

**Affiliations:** † Department of Inorganic Chemistry, 16741 University of Granada, 18071 Granada, Spain; ‡ Department of Organic and Inorganic Chemistry, EPS Linares, University of Jaén, 23700 Linares, Spain; § Department of Material Science and Metallurgy Engineering and Inorganic Chemistry, University of Cádiz, 11510 Cádiz, Spain; ∥ Department of Applied Physics, Faculty of Sciences, University of Granada, 18071 Granada, Spain

**Keywords:** hydrogels, protein fibers, fibrillar polysaccharide, IPN networks, deep learning, real-time CLSM imaging

## Abstract

Fibrillar protein hydrogels are promising sustainable biomaterials for biomedical applications, but their practical use is often limited by insufficient mechanical strength and stability. To address these challenges, we transformed native proteins into amyloid fibrils (AFs) and incorporated a fibrillar polysaccharide, phytagel (PHY), to engineer interpenetrating polymer network (IPN) hydrogels. Notably, we report for the first time the formation of an amyloid-based hydrogel from apoferritin (APO), with PHY reinforcing the network’s mechanical integrity. In situ self-assembly of APO within the PHY matrix yields fully natural, biopolymer-based IPNs. Rheological analyses confirm synergistic interactions between AF and PHY fibers, with the composite hydrogels exhibiting significantly enhanced viscoelastic moduli compared with individual components. The AF–PHY hydrogels also demonstrate excellent self-healing behavior, rapidly restoring their storage modulus after high-strain deformation. A major advancement of this study is the application of deep learning (DL)-based image analysis, using convolutional neural networks, to automate the identification, segmentation, and quantification of fibrillar components in high-resolution scanning electron microscopy images. This AI-driven method enables precise differentiation between AF and PHY fibers and reveals the three-dimensional microarchitecture of the IPN, overcoming key limitations of traditional image analysis. Complementary real-time confocal laser scanning microscopy, with selective fluorescent labeling of protein and polysaccharide components, further validates the IPN structure of the hybrid hydrogels. Our results demonstrate that DL significantly enhances structural characterization and provides insights into gelation processes. This approach sets a new guide for the analysis of complex soft materials and underlines the potential of AF–PHY hydrogels as mechanically robust, self-healing, and fully sustainable biomaterials for biomedical engineering applications.

## Graphical abstract

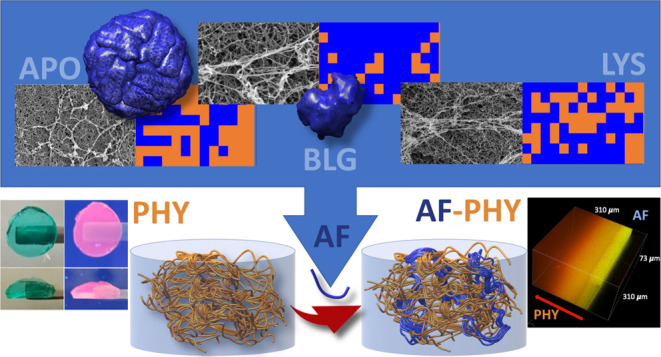

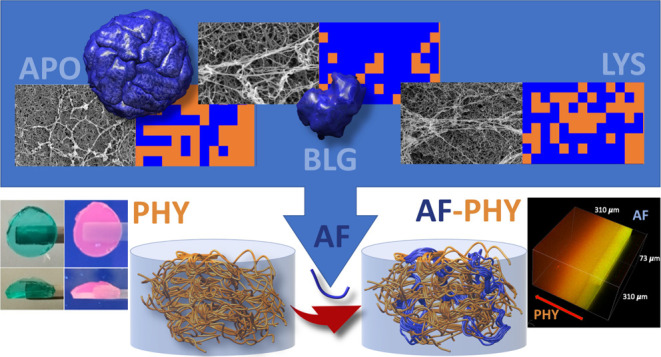

## 1 Introduction

Natural biopolymer-based materials are gaining significant interest as sustainable alternatives to traditional synthetic polymers, particularly in biomedical applications.^1–7^ Synthetic polymers often suffer from biocompatibility and toxicity concerns, making biopolymers (naturally derived polysaccharides, peptides, and proteins) a promising option.^4^ Biomedical hydrogels can benefit from the integration of biopolymers due to their inherent biochemical and biophysical properties, such as bioactivity, degradability, and viscoelasticity.^3^

Despite their potential benefits, biopolymers can also have disadvantages, such as weaker mechanical properties. This limitation can be overcome by enabling cross-linking chemistry with synthetic hydrogel. Indeed, this route has extended the range of achievable properties of biopolymer hydrogels.^8–10^ Polymer blends and composite hydrogel formulations have expanded the range of the physical properties of hydrogels. However, these materials can frequently undergo phase separation. In addition, covalent cross-linking of functional chemical groups may require the use of toxic cross-linking agents and harsh chemical conditions, resulting in the formation of harmful byproducts.^11^ This is not consistent with the current global sustainability framework.

The concept of IPN technologies, which have been developed for synthetic hydrogels, has provided a foundation for the creation of biopolymer-based IPNs.^11–16^ These are formed by the combination of multiple independent polymer networks at the molecular level, resulting in the formation of hydrogels. The individual networks are intermixed but not linked together, with the objective of combining and leveraging the strengths of both networks.^17–19^ This approach enables tuning and improving the mechanical properties of hydrogels.

Proteins are essential components for high-performance materials in nature.^20^ They serve structural purposes, such as silk and collagen, and form active structures, such as the cytoskeleton. This class of molecules is incredibly versatile and is increasingly being explored to synthesize the next generation of green functional materials for various applications.^20–22^ Protein nanofibrils are a truly remarkable supramolecular unit that forms the basis of numerous macroscopic protein materials.^20^ They are one of the best-known examples of materials that can transfer properties from the nanoscale to the macroscopic scale.^23^ Strands and fibers are commonly used as precursors for the production of nanofibrillar gels with higher gel strength and fracture properties, improved water retention capacity, and transparency.^21^

Protein fibril self-assembly offers several intrinsic advantages when used as building blocks to construct a gel network.^20,22^ These include excellent biocompatibility and biodegradability as well as low cell toxicity and immune response. A variety of protein-based hydrogels have been developed utilizing fibrous proteins, including collagen, keratin, elastin, lysozyme (LYZ), and silk fibroin,^24–26^ as well as globular proteins such as β-lactoglobulin (BLG),^27–30^ casein,^31^ serum, and ovalbumin.^20,32,33^ Pioneering work by Mezzenga and co-workers has demonstrated how protein-derived amyloid fibrils (AFs) can serve as functional building blocks for a wide range of applications.^27^ For example, AFs from BLG have been employed as stabilizers in emulsions, carriers for nutraceuticals and pharmaceuticals, and biocompatible scaffolds for tissue engineering.^27,28^ Furthermore, these systems have been explored for use in food structuring, water purification, and biosensing, illustrating their potential across a range of applications.^28–30^ However, the majority of fibrillar protein-based hydrogels exhibit relatively weak mechanical properties, which are drawbacks for some applications. To enhance these properties, a common strategy is to incorporate fillers and cross-linkers such as nanocomposites, surfactants, and polymers into the protein structure, forming a single-network structure.^34^ However, while this approach can improve the mechanical properties of the hydrogel, it often falls short of the strength observed in strong hydrogels. To overcome this issue, double-network structures have been recently fabricated,^22,35^ including nonsustainable protein-synthetic polymer blends or cross-linked networks.^19^

In light of the favorable background and the identified gap, we propose an innovative approach that combines the concepts of IPN and protein self-assembly to fabricate sustainable IPN protein–polysaccharide fibrillar hydrogels using a simple two-step procedure with excellent chemical functionalization and mechanical properties.

For the fabrication of the protein polymeric network, we have used three proteins in their AF form as starting materials: the apoferritin protein (hereafter APO),^36^ one of the main proteins involved in the homeostasis of iron in living organisms,^37^ BLG,^38^ an inexpensive milk protein isolated from whey protein and LYS^39^ extracted from hen egg white, a lytic enzyme with great biocompatibility. The structural integrity of the protein-based hydrogels is enhanced by the second polymer forming the IPN, the polysaccharide PHY (high-acetylated gellan gum). PHY is a water-soluble anionic exopolysaccharide and a gelling agent. This polysaccharide has high temperature stability, high mechanical strength, and great transparency. Structurally, it is made up of a repeating tetrasaccharide unit consisting of two glucose, one rhamnose and one glucuronic acid.^40,41^ The high- and low-acetylated gellan gum forms have been previously used as reinforcing materials.^18,42–44^

The design of biopolymer-based hydrogels combining protein AF s and polysaccharides presents a promising strategy for developing materials with enhanced mechanical and self-healing properties. Among such systems, IPNs formed by protein and polysaccharide components can benefit from synergistic behavior, resulting in improved structural performance and functionality. However, developing such materials is challenging because it is difficult to accurately differentiate the two protein and polysaccharide blocks and visualize their arrangement inside the IPN structure, an essential aspect for understanding their properties. Indeed, in our system, both AF and PHY exhibit diffuse and intertwined morphologies at the nanoscale, making it difficult to distinguish between the two based solely on differences in electron density or structural features. Conventional scanning electron microscopy (SEM) techniques often lack the necessary contrast or resolution to definitively identify and delineate the individual AF proteins and PHY domains within such complex IPN architectures.

The inherent limitations of directly identifying AF and PHY in our system, as well as identifying the two blocks in other protein–polysaccharide IPN materials, necessitate the adoption of more advanced characterization strategies. In this context, artificial intelligence is increasingly offering novel tools in materials science to extract deeper insights from complex structural data.^45^ Among these tools, deep learning (DL) techniques, particularly convolutional neural networks (CNNs), have emerged as powerful methods for interpreting image-based data sets.^46,47^ DL algorithms can be trained to recognize subtle variations in texture, morphology, and spatial organization that are difficult to detect through conventional image analysis. When applied to high-resolution electron microscopy images, CNNs can learn to differentiate between overlapping protein and PHY fiber domains by identifying features specific to each network component.^48^ This approach enables automated, high-fidelity segmentation and classification of biopolymeric networks, offering a significant advancement in the structural characterization of IPN hydrogels.

This report presents two novel findings: first, the formation of an APO protein-based hydrogel and, second, the preparation of hybrid IPN hydrogels by in situ protein polymerization within a previously PHY-formed hydrogel. The APO protein-based hydrogels’ structure and rheological properties are compared to those of other amyloid protein-forming hydrogels, specifically BLG and LYS. A key highlight of this work is the integration of advanced imaging techniques and DL for structural analysis. High-resolution SEM (HRSEM) combined with CNNs enabled precise differentiation between AF and PHY fibers, while confocal laser scanning microscopy (CLSM) using dye-functionalized protein and PHY confirmed the IPN nature of the hydrogels. Additionally, the AF–PHY hydrogel system demonstrated remarkable self-healing properties, with its storage modulus fully recovering within seconds after high strain.

## 2 Materials and Methods

### 2.1 Preparation of APO, BLG, and LY Stock Protein Solutions

Horse spleen from APO protein, BLG from bovine milk protein, and LYZ from hen egg white protein were purchased from MERCK LIFE SCIENCE SLU. APO (3 mL, 3 wt %), BLG (3 mL, 2.7 wt %), and LYS (3 mL, 5 wt %) protein solutions were filtered through a 0.22 μm Millipore filter.

### 2.2 Preparation of APO, BLG, and LY (AF) Hydrogels

The purified protein solutions were adjusted to pH 2 (1 M HCl, 15 min for BLG and LYS and 1 h for APO) before heat treatment (90 °C and 100 rpm, in hermetically sealed glass tubes) for a specific incubation time (APO 24 h, BLG 3 h, and LY 12 h). After heat treatment, the glass tube was cooled in an ice bath to quench the aggregation process of the amyloid fibers (AFs).

### 2.3 Preparation of PHY Hydrogel Discs

Phytagel (PHY) BioReagent was purchased from MERCK LIFE SCIENCE SLU. A 100 mL PHY solution (1.5 wt %) was autoclaved (16 min, 121 °C) and poured into Petri dishes. PHY discs were cast by using a 1.2 cm diameter template.

### 2.4 Preparation of Hybrids AF–PHY Hydrogels

The purified protein solutions were adjusted to pH 2 (1 M HCl, 15 min) and then transferred to a hermetically sealed glass tube. A PHY disc was introduced into the glass tube 15 min before the heat treatment (90 °C, 100 rpm, with appropriate incubation time for each protein). The glass tube was then cooled in an ice bath to quench the protein aggregation process. The disc was subsequently removed from the glass tube and washed with Milli-Q water for 24 h.

### 2.5 Preparation of ATTO488-AF Protein Hydrogels

To prepare the ATTO488-functionalized protein hydrogels, the purified protein solutions and ATTO488 maleimide solution (20 μL, 2 mg/mL) were mixed for a period of 15 min. Subsequently, a heat treatment was performed in hermetically sealed glass tubes (90 °C and 100 rpm, at adequate incubation time for each protein) in the absence of light. The glass tubes were then cooled in an ice bath to quench the protein aggregation process.

### 2.6 Preparation of ATTO647-PHY Hydrogel Discs

1-Ethyl-3-[3-(dimethylamino)­propyl] carbodiimide hydrochloride (EDC) was purchased from Merck. A PHY disc was placed in a hermetically sealed glass tube containing 3 mL of Milli-Q water. A solution of 6 mg/mL EDC was prepared, and 10 μL was added to the glass tube to activate the polysaccharide carboxyl groups. After 30 min, a stock solution of ATTO647 amine (10 μL, 2 mg/mL) was added. This was followed by heat treatment (90 °C, 100 rpm, 3 h). The disc was then removed from the glass tube and washed thoroughly with a Milli-Q water solution for 24 h.

### 2.7 Preparation of ATTO488-AF/ATTO647-PHY Hybrid Hydrogel Discs

A solution containing the fluorophores ATTO488 and ATTO647 (in a 1:5 ratio) and the corresponding purified protein was added to a hermetically sealed glass tube. A PHY disc (previously treated with EDC) was placed in the glass tube and allowed to react for 30 min prior to heat treatment (90 °C, 100 rpm, with appropriate incubation time for each protein). The glass tube was then cooled in an ice bath to quench the AF aggregation process. The disk was then removed from the glass tube and washed with Milli-Q water for 24 h.

### 2.8 Samples for Dynamic Light Scattering and Zeta Potential

The values of the average hydrodynamic diameter dynamic light scattering (DLS) and the zeta potential (ZP) of protein prehydrogels (dilution 50 in pH2Milli-Q water) were obtained by using a Particle Analyzer Litesizer 500 (Anton Paar) at 25 °C. The polydispersity index of the samples showed values around 20–25%.

### 2.9 Fourier Transform Infrared Spectroscopy

Fourier transform infrared spectroscopy (FTIR) spectra were obtained by using a Bruker Tensor 27 FTIR spectrometer. Sample tablets (200 mg KBr + 2 mg aerogel sample) were scanned from 4000 to 400 cm^–1^ with a resolution of 4 cm^–1^ at room temperature and averaged over 16 scans. Spectra were background subtracted and smoothed by using the OPUS Data Collection Program.

### 2.10 Samples for HRSEM

Aerogel samples were prepared as follows: fixation of the samples in a 2.5% glutaraldehyde solution in water, for 24 h and at 4 °C. Washing of the samples in water containing the fixative (3 changes of 20 min at 4 °C). Dehydration in a gradient of increasing concentrations of MERCK ethanol (50%, 70%, 90%, and 100%). Drying by the Critical Point Method (Anderson, 1951) was done with carbon dioxide in a Polaron CPD7501 Critical Point Dryer. Coating of the samples with platinum 3 nm (EM ACE 600). The samples were observed with an AURIGA (FIB-FESEM) de Carl Zeiss SMT Scanning Electron Microscope and a High Resolution Variable Pressure Scanning Electron Microscope (FESEM) Zeiss SUPRA40VP.

### 2.11 Confocal Microscopy Measurements

The fluorescence of ATTO488-AF, ATTO647-PHY, and ATTO488-AF/ATTO647-PHY hydrogels discs was evaluated using a Leica TCS-SP5 II Confocal microscope equipped with a 25× 0.95NA water immersion objective. The hydrogel discs were sectioned prior to analysis as shown in the figure. After being sectioned, the protein samples were placed on glass coverslips for image acquisition. Argon (488 nm) and HeNe (633 nm) laser lines were used sequentially to acquire ATTO signals (green and far-red channels). Images were acquired using an xyz scan mode, 512 × 512 px resolution, and 400 Hz scan speed. PMT detectors were used to detect the fluorescence emission of the samples. (Detection bands were set at 500–550 and 640–740 nm for the green and far-red channels, respectively.) The pinhole size was set to 1 AU for all measurements. Maximum intensity projections were obtained by using Leica LAS AF software, and 3D rendering was performed by using NIS Elements (Nikon) and ImageJ Fiji software.

### 2.12 Rheological Characterization of Hydrogels

A rheometer (MCR302, Anton Paar) controlled by Rheoplus/32 software (Multi3 V3.62 21006097-33028) was used to investigate the mechanical properties of the hydrogels. The experiments were performed at 25 °C in a parallel plate configuration (20 mm diameter, PP20/MRD/TI/P2/CUST-SN64538). Plates with rough surfaces were used to prevent sample slippage. All rheological experiments were performed in triplicate for each sample. The linear viscoelastic response of the protein and PHY-protein hydrogels was performed by strain sweep measurements, which were tested by applying strains of 0.01%–100% and 0.01–1000%, respectively, at constant frequency (*f* = 1 Hz). The gap was fixed at 0.8 mm. Frequency sweep experiments were constructed by applying a frequency from 0.1 to 100 Hz at constant strain amplitude (0.1%). The gap was fixed at 0.8 mm. The viscosity properties of the hydrogels and PHY-protein hydrogels were measured for a range of shear stress in the intervals 0.1–1000 Pa and 10–5000 Pa, respectively. The gap was fixed at 0.8 mm. The self-healing properties of the hydrogels were measured by continuously applying high (100%) and low (0.1%) oscillatory strain amplitude at a constant frequency (*f* = 1 Hz) to test the mechanical recovery performance. The gap was fixed at 0.8 mm. A compression test was performed to determine Young’s modulus. The specimen was placed on the lower plate using forceps, and the upper plate was moved downward (i.e., closing the gap) at a constant speed (υ = 10 μm/s). During plate movement, the normal force applied to the plate by the sample was monitored. The top plate stopped when the normal force (FN) reached a value of 0.3 N.

### 2.13 Nitrogen Adsorption Experiments

Nitrogen gas adsorption was performed using a Tristar 3000 instrument (Micromeritics) at 77 K. Prior to measurements, the samples were degassed for 3 h at 60 °C under a continuous nitrogen flow. The Brunauer–Emmett–Teller (BET) method was used to determine the specific surface area of the aerogels.

### 2.14 Elemental Analysis

Elemental analysis was performed on a Fisons-Carlo Erba model EA1108 analyzer.

### 2.15 Width Fiber Measurements from SEM Images

An automated image processing pipeline was used to measure the width of each fiber in the SEM images. This pipeline included several steps: segmentation: the first step was to separate the fibers from the background in the SEM images. This process, called segmentation, uses image intensity to distinguish the fibers (brighter pixels) from the background (darker pixels). The result is a binary image, where pixels representing fibers are assigned a value of 1 and background pixels are assigned a value of 0. Distance transform: next, a Euclidean distance transform map was created based on the binary image. This map assigns each pixel a value representing the distance to the nearest fiber edge. Skeletonization: the distance transform map was then processed by using a technique called skeletonization. This process simplifies the representation of the fibers by thinning them down to a single-pixel wide centerline. Centerline refinement: finally, morphological operations involving dilation and erosion were applied to the skeletonized image. These operations refine the centerline by removing any remaining irregularities and ensuring that it accurately represents the center of the fiber. Width measurement: the final, refined centerline was used to calculate the local width along the fiber. The local width value is mapped to a color palette.

### 2.16 DL Technique Applied to SEM Images

To differentiate between AF protein fibers and PHY polysaccharides within AF–PHY hydrogels, we used a DL technique based on a CNN. Specifically, we used the pretrained ResNet-50 architecture, a powerful tool for image recognition (Chen, K. and Barnard, A. S. Advancing electron microscopy using DL. *J. Phys. Mater.* **2024,** *7,* 022001). Since CNNs require labeled training data, we used experimental SEM images of both protein fibers and pure PHY hydrogels taken at different magnifications. To create a suitable training data set, these images were cropped into smaller 224 × 224 pixel squares using a sliding window approach, resulting in a data set of 1600 images. To ensure effective training and to avoid overfitting, the data set was divided into two subsets: a training set (80%) and a validation set (20%). The training set was used to train the CNN to recognize patterns in the images and accurately classify protein fibers and PHY. The validation set was used to evaluate the performance of the model on unseen data, allowing us to fine-tune the training process. To conduct a more in-depth analysis of our DL model’s performance under these training conditions, a confusion matrix was generated (see Figure S8). Based on this confusion matrix, the ResNet50 model has achieved exceptional performance on the validation set, successfully classifying the five distinct classes in both cases, which illustrates a very strong performance for the ResNet50 model in classifying these biological categories. However, it is vital to consider the potential for overfitting. This phenomenon might occur if (i) the training and validation data sets exhibit a high degree of similarity and (ii) the model’s complexity is disproportionately high relative to the volume of available data. In this regard, we also tested its performance on SEM images of pure BLG, APO, PHY, and LYS hydrogels, which were not used for either the training or validation step. These images were also cropped by using the same approach as the training data. Each crop was fed into the CNN as an input image, resulting in a prediction score for each fiber type (all AFs and PHY). The maximum prediction score was used to determine the most likely classification. This classification information was then used to create a color-coded map. Figures S9 and S10 show the original SEM image along with the color-coded map generated by the CNN. Note that the model (i) scores the presence of PHY regions as 0%, despite the nature of the AFs, indicating that AFs are clearly distinguished from PHY fibers. This must be related to clear differences in the fine details of the feature contrasts of both types of fibers, which are very likely to be overlooked in a direct eye observation; (ii) accurately classifies APO hydrogels, achieving over 95% accuracy for both BLG and LYS; (iii) the small percentage of misclassifications (<5%) in the case of LY are due to the assignment to BLG, most likely due to the close resemblance in morphology of these two types of AFs. However, the overall recognition rate is high, demonstrating the ability of the CNN to correctly identify protein types. Therefore, the algorithm can be applied to the ultimate goal, which is the identification between the fibers and the PHY.

## 3 Results and Discussion

APO globular protein forms 3D macroscopic hydrogels when present at appropriate concentrations and subjected to denaturing conditions, that is, a thermal treatment at a temperature in between 50 °C and 90 °C and for an incubation time of 24 h–48 h (Figure S1). As an example, a pregel stable protein solution (3% w/v, pH 2) containing small and large oligomeric peptides (as determined by DLS) (Figure S2), was heated at 70 °C for 24 h (Figure 1). The presence of both small and large oligomeric species ensures a dynamic equilibrium that supports continuous fibril growth and network formation. The solution pH, relative to the peptide’s isoelectric point (pI) or the p*K*_a_ of key residues, strongly influences aggregation behavior by modulating electrostatic interactions and solubility, which in turn affects the size and distribution of oligomers available for gelation.^49^ The hydrogel formed almost instantaneously; longer incubation times were required for obtaining a fibrillar network structure over the entire sample. Heating the APO protein in acidic conditions (pH ∼ 2) between 50 and 90 °C induces partial denaturation, unfolds the native globular APO structure, and exposes hydrophobic and β-sheet-prone regions. As a result, small and large subunit peptides undergo structural rearrangement and self-assembly into AFs through a nucleation-dependent mechanism.^36,49^ Protein concentration and temperature are the key factors triggering the gelation process. Gelation is induced even at low temperatures (50 °C) and low ionic strength media (60 mM NaCl). APO nanofiber hydrogels (Figure 1A) were compared with other hydrogel-forming globular proteins, such as BLG and LYS (Figure 1C,E). Typically, BLG and LYS hydrogels are prepared following a multistep approach, starting from isolated rigid fibrils that gel upon medium salt alteration. In contrast, APO hydrogels fabricated in this work are prepared following a single-step approach by simply heating the protein at a pH far from its isoelectric point (=4.5).

**Figure 1:**
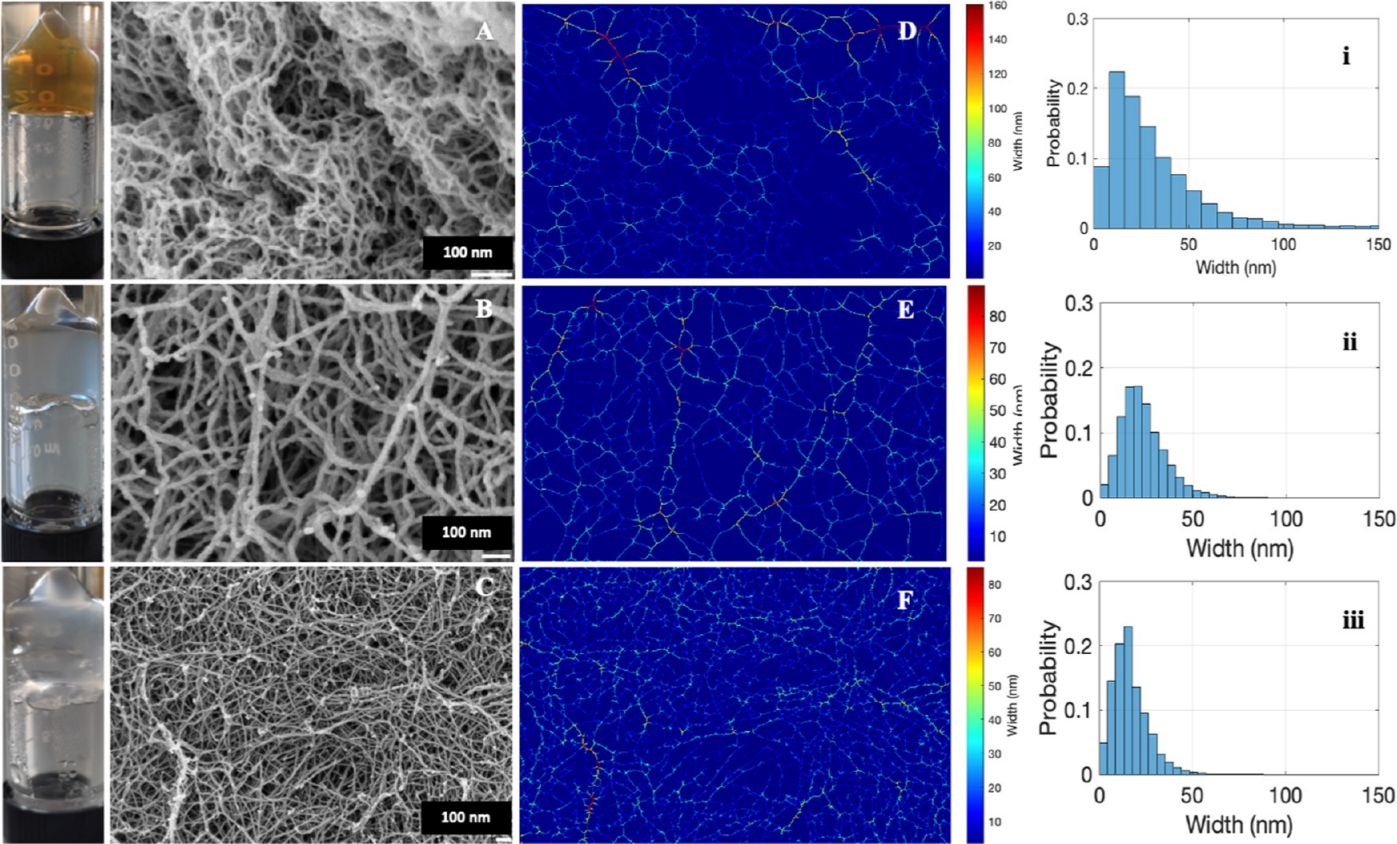
(A–C) FESEM images and (D–F) corresponding analysis images and (i–iii) histograms of APO, BLG, and LYS hydrogels, respectively. Macroscopic images of self-standing hydrogels are also included (A–C inset).

Photographs of the macroscopic appearance of pure protein hydrogels are shown in Figure 1A–C (inset). Their transparency is immediately appreciated, together with their self-standing properties using the inversion tube test. The FESEM images (Figure 1A–C) revealed networks with mainly single fibers, forming the backbone of the network for transparent aerogels. By optimizing the initial protein content (≥2.5% wt) and temperature (≥50 °C), a 3D scaffold with a pore size of up to a few microns and high porosity was produced. Morphologically, it is clear that the starting fibrillar morphology of the amyloid protein is preserved. To quantify the local width along the fibers, an automated methodology was employed, as outlined in the Supporting Information. This approach facilitated an efficient analysis that was both unbiased and statistically significant. The fiber width distribution for each sample is shown in Figure 1D–F. These figures reveal a broader width range for APO fibers, spanning from 10 to 160 nm, in comparison to those of BLG and LYS fibers. BLG and LYS fibers exhibit a narrower distribution, falling within a range of 10 to 90 nm. These observations are further substantiated by the histograms presented in Figure 1(i–iii). This broader distribution likely arises from the structural characteristics of APO. Unlike the smaller monomeric BLG (18.4 kDa) and LYS (14.3 kDa), APO is a large multimeric protein (∼500 kDa) composed of 24 subunits. Under acidic and thermal treatment, APO undergoes partial disassembly into heterogeneous subunits that subsequently self-assemble into fibrils. These conditions promote diverse nucleation events and lateral associations, leading to fibrils with a wider range of widths and degrees of bundling. This heterogeneity reflects greater flexibility in self-assembly dynamics and suggests that APO fibrils can form more complex and interconnected morphologies than those derived from BLG and LYS. In order to characterize the overall fiber width distribution for each sample, the histograms were clustered into three categories that were representative of the data: thin, medium, and thick segments. This clustering approach reflects the observed fiber agglomeration in the SEM images, where some fibers appear clumped together. The width ranges are represented visually in Figure S3 by using a three-color map. This color map effectively highlights the distribution of fiber widths within the SEM images. The analysis of these distributions enabled the determination of the average fiber widths, employing the medium segment cluster. The values for APO, BLG, and LYS were 20 ± 2 nm, 21 ± 2 nm, and 19 ± 1 nm, respectively.

### 3.1 Mechanical Properties of Pure AF Hydrogels

Tunable mechanical properties are a fundamental requirement for hydrogels when considering practical applications. Therefore, the mechanical properties of pure APO amyloid-based hydrogels were investigated by rheological measurements and compared with those of BLG and LYS (Figure S4). Like many other biological materials, amyloid-based hydrogels exhibit viscoelastic behavior.^39,50^ The storage modulus *G*′ is higher than the loss modulus *G*″ over the entire strain amplitude or frequency range studied for the three amyloid protein hydrogels, confirming a solid-like behavior typical of gels (Figure S4A). Typically, the *G*′ value is correlated with the network strength. The storage modulus *G*′ of APO hydrogels is comparable to that of BLG and moderately higher than that of LYS hydrogels. This enhanced modulus can be attributed to the structural features of the APO-derived fibrils. The multimeric nature and higher molecular weight of APO allow the formation of fibrils with broader width distributions and the potential for lateral bundling. These thicker, more flexible fibrils create a denser and more interconnected network with a greater number of load-bearing junctions, thereby increasing the overall stiffness and resistance to deformation. The viscoelastic moduli are frequency independent, and the terminal region is not observed in the mechanical spectrum (Figure S4B). The damping factor (tan δ = *G*″/*G*′) for the three pure proteins is below one and ranges from 0.1 to 0.3, so we can conclude that the hybrid hydrogels form stable structures (Figure S4C). The shear strength (i.e., the maximum in elastic shear stress) was approximately 3× higher for APO hydrogel compared to that of LYS or 15× higher compared to the BLG hydrogel (Figure S4D). Moreover, the strain amplitude corresponding to the shear strength was also higher for APO hydrogel. These findings indicate that APO fibers exhibit a high degree of flexibility under low shear strain, with minimal impact on the *G*′ and *G*″ values corresponding to the viscoelastic regime. At larger shear strains, within the nonlinear viscoelastic regime, the resistance to deformation is notably improved.

Although pure APO forms hydrogels with stiffness and strength comparable to or larger than those of BLG and LYS, the limited mechanical strength of these pure protein hydrogels is a potential limitation if they are subjected to considerable strain and stress. We therefore decided to reinforce pure APO hydrogels by preparing hybrid IPNs using the highly acetylated gellan gum polysaccharide PHY, a well-known gelling agent. For a comparative purpose, we also prepared the corresponding IPNs using BLG and LYS proteins with the aim of establishing correlations between the protein nature and the structure and mechanical properties of the IPNs.

### 3.2 In Situ Protein Self-Assembly within PHY Hydrogels to Form Interpenetrated Polymer Networks

The in situ diffusion and formation of protein fibers within a previously prepared polysaccharide fibrillar hydrogel represents a novel and promising synthetic approach that could result in protein assembly materials with superior mechanical and chemical properties compared to those of homologous biomaterials made with the same components but in their native form or by simple mixing of their constituents. Therefore, for the preparation of the IPN hydrogels, first, a PHY hydrogel disc is cast and introduced into a solution containing small peptides of the corresponding protein (Scheme 1) and after an appropriate heat treatment, the disc is removed and its structure thoroughly studied by HRSEM.

**Scheme 1:**
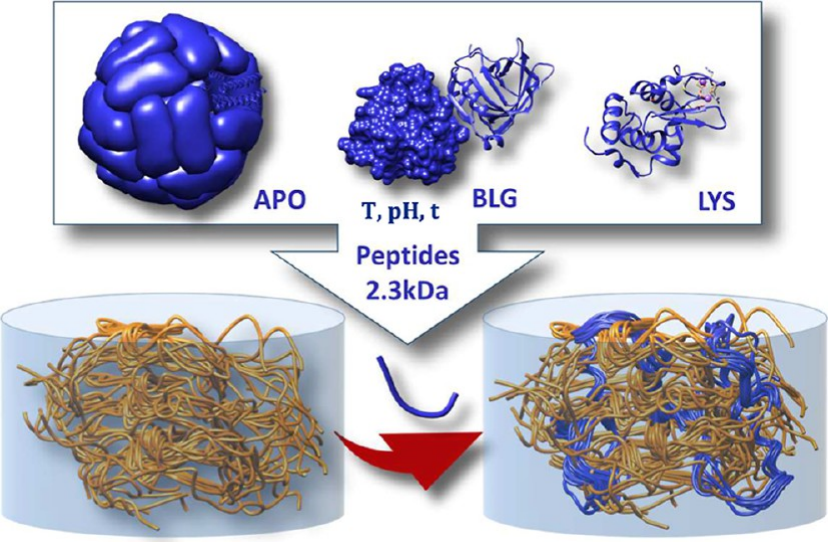
Schematic of the Strategy for Producing Versatile Materials through AF In Situ Polymerization within PHY to Form IPNs

Thus, amyloid protein-PHY hybrid hydrogels (AF–PHY; AF stands for amyloid fibers of APO, BLG, or LYS) are obtained by the formation and subsequent diffusion of the small peptides into the nanoconfined porous structure of the PHY disc, leading ultimately to the formation of the IPN. It is important to note that under pregel conditions, biocolloids exhibit very low surface charges of opposite sign, as measured by ZP (Figure S5), which avoids strong ionic interactions and thus aggregation by complexation.

Specifically, a 1.5% w/v PHY solution was autoclaved at 120 °C for 1 h, and a disc was cast on cooling. The drop in the temperature triggers a coil-to-helix transition in the PHY molecules, resulting in a 3D gel structure. At high temperatures, PHY chains adopt random coil conformations; as the temperature decreases, they reorganize into ordered double helices. These helices further aggregate into junction zones that act as cross-linking points, creating a continuous network. This process is stabilized by hydrogen bonding between hydroxyl groups, hydrophobic, and ionic interactions that shield repulsive charges and promote helix aggregation.^40,42,43^ The PHY disc was then immersed in a sealed glass bottle containing the APO protein solution at pH 2 and incubated at 50 °C–90 °C for 24–48 h. In the case of BLG and LYS proteins, the reaction temperature was 90 °C, and the incubation times were 3 and 12 h, respectively. Figure 2 illustrates the self-supporting nature of the three discs of the AF–PHY IPNs. Due to the highly porous PHY structure, the protein self-assembly process takes place within the PHY network, forming a hybrid fibrillar hydrogel. The protein-occupied 3D fibrillar percolation PHY structure provides superior mechanical properties, clearly visible to the naked eye.

**Figure 2:**
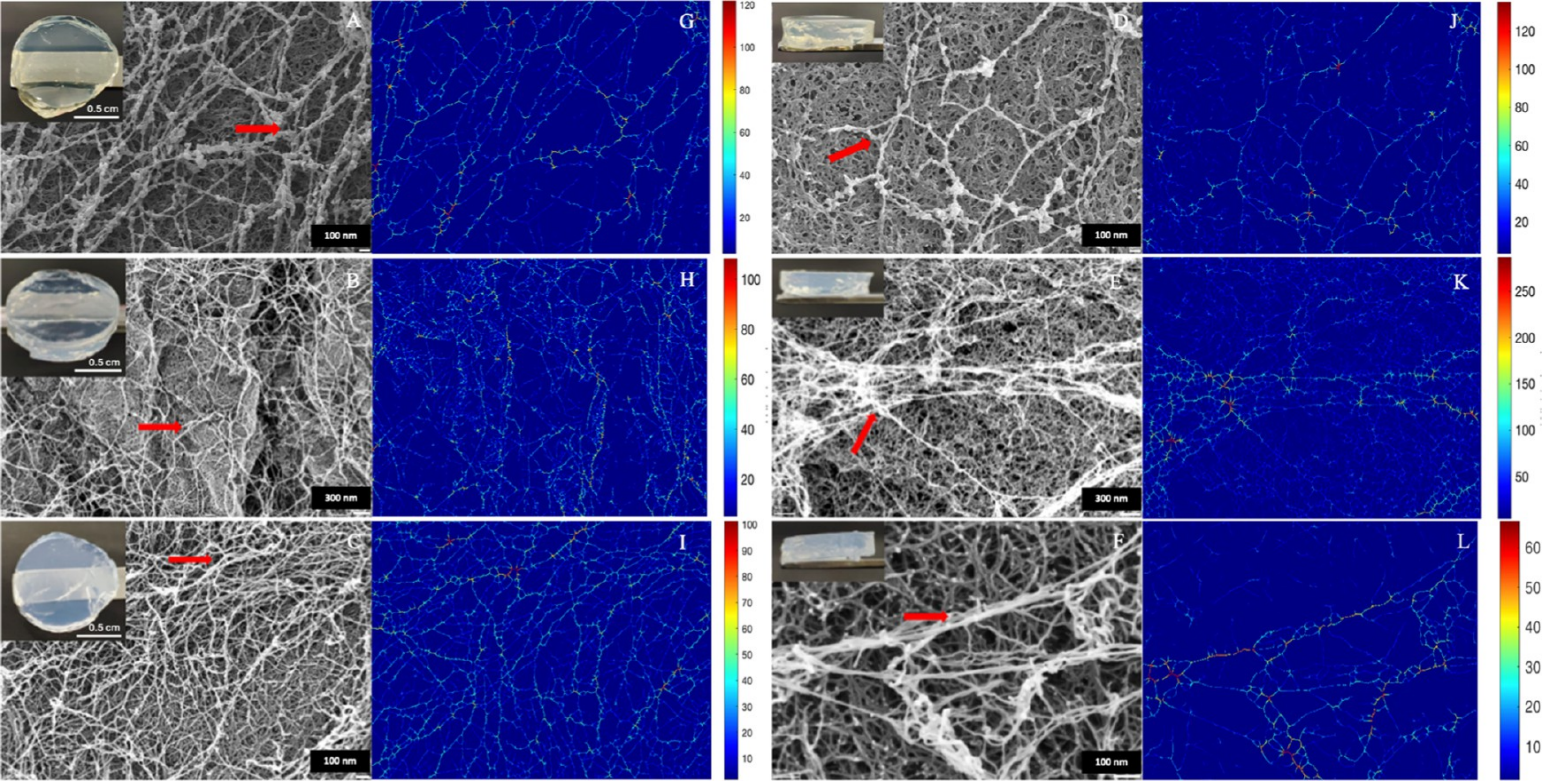
(A–C) Hydrogel disc exterior HRSEM images and (D–F) hydrogel disc interior HRSEM images of APO-, BLG-, and LYS-PHY IPN hydrogels, respectively. The red arrows highlight the brighter protein fibers. Macroscopic images of hydrogel discs are also included (insets). (G–L) Corresponding analysis images.

The high-resolution SEM images of the exterior and interior of the AF–PHY hydrogel discs (Figure 2A–C and 2D–F, respectively) reveal in all cases open and highly porous structures within which two types of nanofibers appear to be distinguishable. The amyloid protein fibers, indicated by red arrows, are likely the brighter ones, in contrast to the darker, denser matrix fibers, which are presumably to represent PHY. Using the same methodology previously conducted to measure fiber widths of the pure AF, the local width maps for AF–PHY hybrids were also estimated, Figure 2G–L. Note that these maps do not reveal significant distinction in diameter between the AF and PHY fibers. After clustering these maps (Figure S6G–L), the mean fiber widths were measured as 28 ± 1, 22 ± 2, and 17 ± 0.5 nm for APO-PHY, BLG-PHY, and LYS-PHY, respectively. SEM images of pure PHY hydrogel (Figure S7) showed a fibrillar structure with a main population of roughly a 15 nm average width.

### 3.3 DL Technique Makes It Possible to Differentiate between the AF and PHY Fibers

To differentiate between AF and PHY within the AF–PHY hydrogels, we employed a CNN DL technique. Specifically, we leveraged the pretrained ResNet-50 architecture, a powerful tool for image recognition. As CNNs require labeled training data, we utilized experimental SEM images of pure both protein fibers and PHY-hydrogels recorded at various magnifications (Figures S9 and S10). To create a suitable training data set, these images were cropped into smaller 224 × 224 pixel patches using a sliding window approach, resulting in a data set of 1600 images. For further details on the training process, please refer to the Supporting Information. It is important to note that in situ polymerization within PHY can alter the structure of the fibers. This modification might hinder accurate classification of specific fiber types. Nevertheless, CNN can still be used to distinguish between the presence of protein and PHY components in SEM images of AF–PHY hydrogels.

Figure 3 illustrates SEM images of the hydrogel disc exterior (Figure 3A–C) and interior (Figure 3D–F), along with the corresponding color-coded maps generated by the CNN. These color-coded maps were obtained by cropping the SEM images into smaller sections and feeding each section into the CNN. The CNN then assigned a prediction score to each crop, indicating the likelihood of it being either an AF protein (magenta) or a PHY polysaccharide (blue). The maximum prediction score was used to determine the most likely classification. This classification information was then used to create a color-coded map. From these maps, we can clearly observe the presence of AF in both the exterior and interior regions of the hydrogel disc. However, the exterior region exhibits a significantly higher concentration of AF, which aligns with the findings of the confocal microscopy study discussed later.

**Figure 3:**
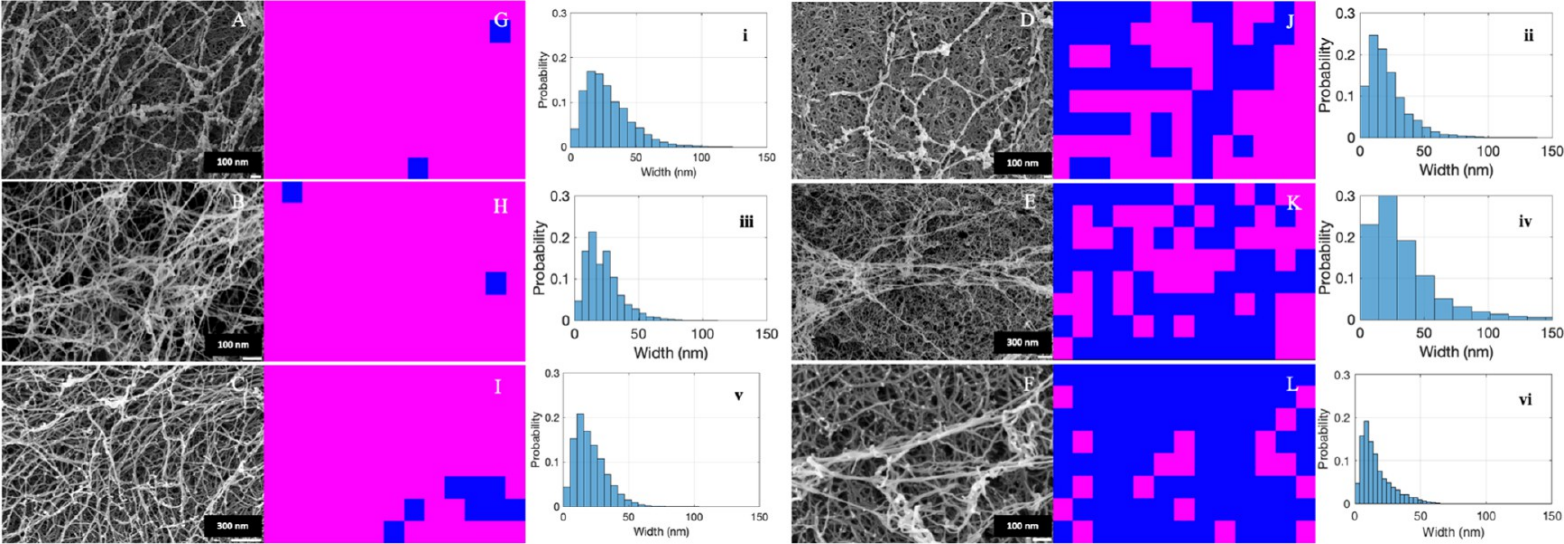
(A–C) Hydrogel disc exterior SEM images and (D–F) hydrogel disc interior SEM images of APO-, BLG-, and LYS-PHY IPN hydrogels, respectively. (G,–L) CNN analysis images. Blue color: PHY and magenta color: AF; (i–vi) the corresponding histograms are also shown.

### 3.4 Revealing the Nanoporous Nature of AF–PHY Hybrid Hydrogels

One limitation of in situ diffusion may come from the relative size of the protein oligomers compared with the average mesh size of the polysaccharide network. Our previous results^45^ demonstrate that APO protein (as well as BLG^51^ or LYS^52^) oligomers are sufficiently small to be able to diffuse into the PHY meso- and macropores. Nitrogen adsorption–desorption measurements were performed at −196 °C for analyzing the pore structure and surface area. Figure 4A shows the nitrogen adsorption–desorption isotherms of the AF–PHY hydrogels. These types of isotherms are characteristic of mesoporous materials with their typical pore diameters between 2 and 50 nm (Figure 4A). BET surface area values range from 205 m^2^/g for APO-PHY and 210 m^2^/g for LYS-PHY to 478 m^2^/g for BLG-PHY. The AF–PHY hybrids show decreasing nitrogen adsorption in the whole relative pressure region compared to pure proteins (Figure S11). These differences reflect the intrinsic properties and assembly behavior of the incorporated proteins. APO fibrils, with their broader width distribution and greater morphological heterogeneity, tend to form denser and more entangled networks that partially occlude the pores within the PHY scaffold, reducing the accessible surface area. In contrast, BLG and LYS fibrils, being thinner and more uniform, preserve more of the mesoporous structure, resulting in higher surface areas. This demonstrates how protein fibril architecture directly influences the porosity and internal surface characteristics of the resulting IPN hydrogels. Figure 4B shows the pore size distributions of PHY and the AF–PHY hybrids, which were calculated based on the density functional theory. Clearly, the mesopore volumes decrease with the increasing amount of in situ protein fiber polymerization. The possible pore size distribution is the reduction first of the smallest mesopores, possibly based on stronger interactions. The results obtained in this BET study support a combination of filling mechanisms: agglomeration of protein oligomers, mesopore filling, and layer filling models.^53^ The successful formation of hydrogels was also probed by FTIR spectroscopy (Figure 4D). The symmetric and antisymmetric COO^–^ stretching bands (1400 and 1600 cm^–1^) typical of amide bonds were observed, together with the broad band of OH stretching in the 3100–3600 cm^–1^ frequency region. The peak at 1150 cm^–1^, which corresponds to the glycosidic bond (C–O–C) of the PHY polysaccharide, was observed in the hybrid hydrogels but not in the pure protein hydrogels (Figure 4D).

**Figure 4:**
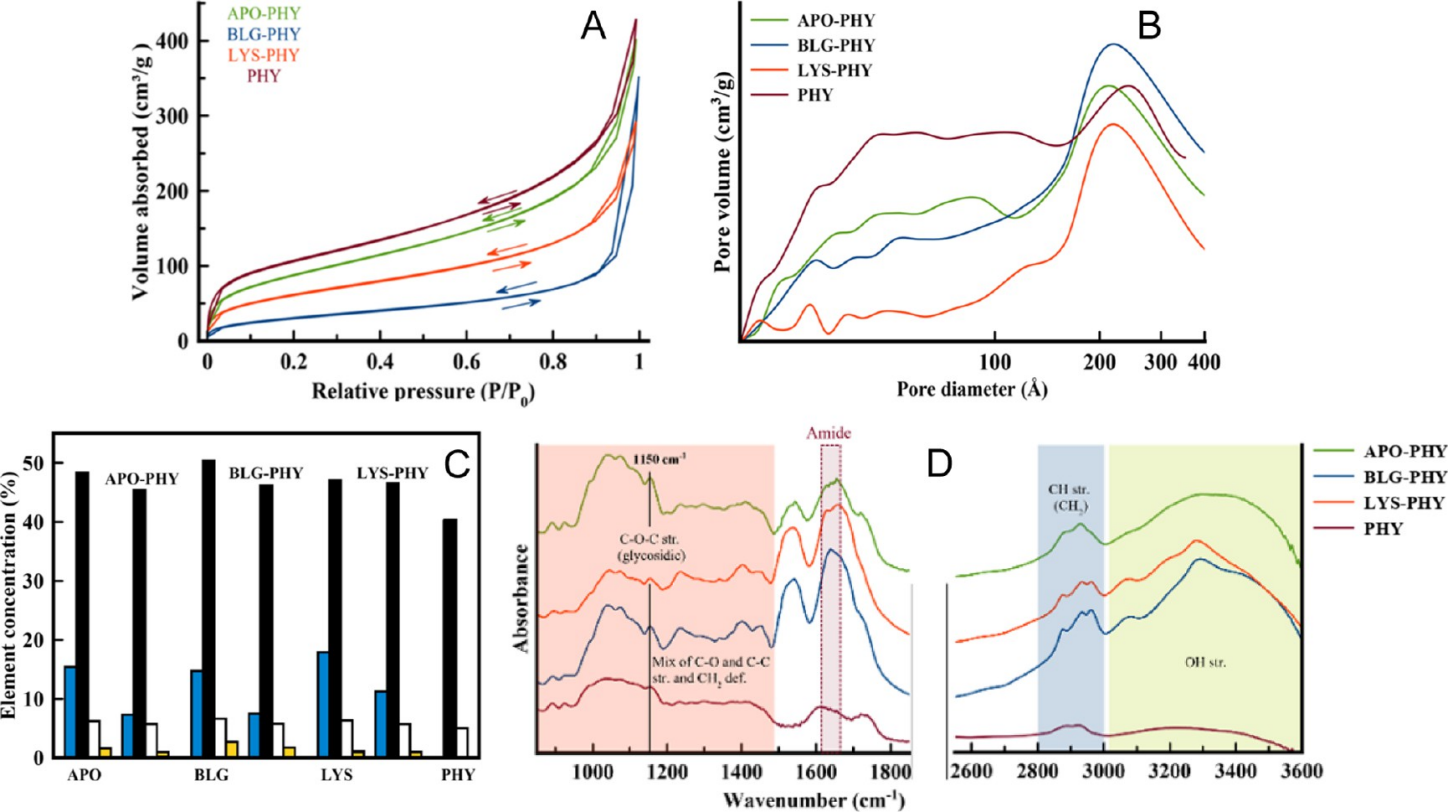
(A) Nitrogen gas absorption–desorption isotherms of the AF–PHY hydrogels. (B) Pore size distribution derived from the nitrogen desorption curves. (C) Elemental analysis of pure protein, pure PHY, and AF–PHY hybrid hydrogels (black: carbon, blue: nitrogen, yellow: sulfur, and white: hydrogen). (D) FT-IR spectra of AF–PHY hybrid hydrogels.

Elemental analysis was performed on pure PHY, pure protein, and all IPN hydrogels. The element concentration was calculated, and the data are shown in Figure 4C. Compared to pure PHY hydrogel, where no nitrogen or sulfur was detected, the pure protein and the IPN hybrid hydrogels contain nitrogen and sulfur, thus confirming the presence of the protein in its structure.

### 3.5 The IPN Strengthens the AF Hydrogels via In Situ Protein Self-Assembly within the PHY Hydrogel

The strength of hybrid AF–PHY IPN hydrogels was measured. The variation of the viscoelastic moduli as well as the damping factor gives insight into the compatibility between the components of the hybrid gels and their ability to form stable networks.^54^ Strain amplitude and frequency sweep measurements were performed (Figure 5A,B). Both experiments confirmed the viscoelastic behavior of the hydrogels. Figure 5A shows the relative viscoelastic moduli in %, taking the moduli of the pure PHY hydrogel as a reference (G-G­(PHY)/G­(PHY)) × 100. It clearly shows that the viscoelastic moduli of the IPNs are 2 orders of magnitude higher than those of PHY and also those of the corresponding pure protein hydrogels (cf. Figure S4), demonstrating a synergistic effect between the protein fiber hydrogel and the PHY hydrogel. Similarly, the frequency sweep tests also confirmed a strengthening of the hydrogel due to the presence of PHY (Figure 5B).

**Figure 5:**
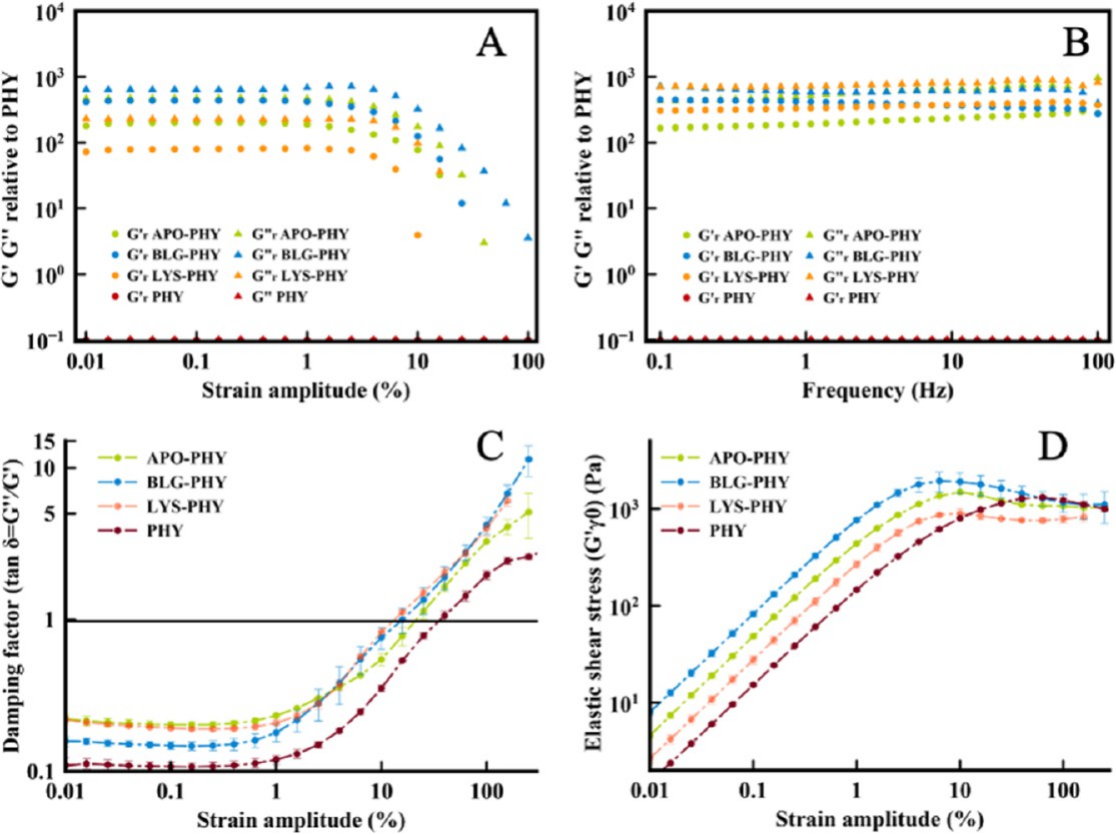
Rheological properties of AF–PHY hydrogels. (A) Normalized strain amplitude sweeps at a constant frequency (*f* = 1 Hz). (B) Normalized excitation frequency sweep at a constant strain amplitude (γ_0_ = 0.1%). (C) Damping factor (tan δ = *G*″/*G*′) as a function of the strain amplitude for the data shown in (A,D) elastic stress (*G*′γ_0_) as a function of the strain amplitude for the data shown in (A).

The damping factor (Figure 5C) for the three AF–PHY IPNs was very similar (close to 0.3 in the viscoelastic linear region), in good agreement with Figure 5A,B, and slightly larger than the one measured for PHY suggesting that the polysaccharide network dominates the response. Again, the shear strength was quite similar for BLG-PHY and LYS-PHY hybrids, while for APO-PHY it is slightly shifted to higher strain amplitude values. Overall, the strain amplitude corresponding to the shear strength was always smaller than that measured with PHY (see Figure 5D). The viscosity measurements of the hydrogels showed a yield stress manifested by a −1 slope in the viscosity curve for pure protein and AF–PHY hydrogels, according to the gel nature of the materials (Figure S12).

Figure 6A shows both the storage modulus measured under shear and the Young’s modulus measured under compression. The Young’s modulus was obtained by measuring the slope of the initial linear region of the stress–strain curve for compressive strains below 0.04. The Young’s modulus of the IPN hydrogels was found to be in the range of 0.9–1.2 MPa, which is approximately 2-fold higher than those of pure PHY and in good agreement with similar gels.^55^ This phenomenon has been attributed to the entanglement enhancement effect, whereby the two intertwined yet separate polymer networks are pulled together by topological constraints.^18,56^ This mechanical stiffness is significantly higher than those of many commonly used natural hydrogels. Young’s moduli for collagen-based hydrogels range from 0.1 to 1.0 MPa, depending on concentration and degree of cross-linking.^57^ Alginate hydrogels generally exhibit moduli between 10 and 100 kPa, though chemical modification or ionotropic cross-linking can increase their stiffness to several hundred kPa.^58,59^ The higher modulus of the AF–PHY hydrogels reflects the synergistic reinforcement provided by the interpenetrating network of AF and PHY fibers.

**Figure 6:**
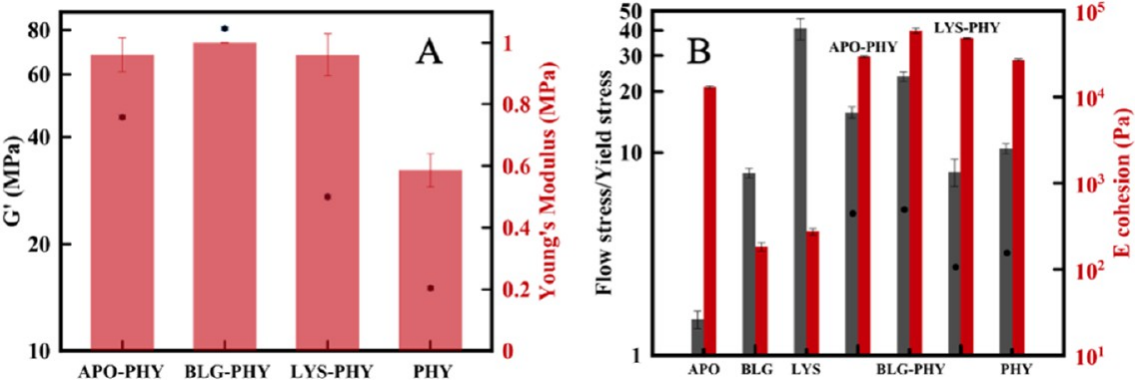
(A) *G*′ (black dots) and Young’s modulus (red bars) of the AF–PHY hybrids as obtained under compression at a compression velocity of 10 μm·s^–1^. (B) Flow stress/yield stress ratio and cohesive energy (*G*′γ_*y*_^2^/2) of AF–PHY hydrogels (black dots: flow stress/yield stress coefficient from viscosity measurements).

Importantly, the mechanical properties of AF–PHY hydrogels fall within the optimal range for mimicking soft biological tissues, including cartilage (∼0.5–1 MPa) and skeletal muscle (∼0.1–1 MPa).^60,61^ This highlights their potential as mechanically robust, tunable, and biocompatible platforms for applications in tissue engineering, including injectable scaffolds, wound-healing matrices, and load-bearing soft tissue replacements.

The left axis in Figure 6B depicts the flow transition index calculated by the ratio between the flow stress (τf, corresponding to *G*′ = *G*″) and the yield stress τ_*y*_. It measures how fast the yielding process occurs; the closer the index is to one, the more brittle the material is. As observed, APO hydrogels are the most brittle among all proteins investigated. However, the associated IPN is the most ductile with the exception of BLG-PHY. For completeness, the flow transition index has also been computed using the yield stress measured in stress sweep ramps in steady shearing flow. Results are shown in dot symbols together with the gray bars exhibiting a good agreement with oscillatory shear data.

The right axis in Figure 6B shows the cohesive energy (*G*′γ_*y*_^2^/2). It is calculated from the strain amplitude sweep tests. Here, γy stands for the yield strain (i.e., the upper strain limit of the viscoelastic linear region). A larger cohesive energy is associated with a better stability of the material. As observed, the most stable hydrogels are clearly the AF–PHY IPNs.

### 3.6 Self-Healing Properties of AF–PHY Hybrid Hydrogels

The biomechanical properties of a material may be influenced by the in vivo conditions under which it is subjected. Alternatively, certain hydrogels exhibit an intriguing property whereby they flow under an applied stress (shear-thin) and readily regain their original stiffness following the removal of the stress (self-healing) without the need for any external stimulus. Shear-thinning hydrogels rely on reversible physical cross-links and include hydrogels made with self-assembling peptides. Remarkably, in step-strain experiments (Figure 7) where a high strain (100%) was introduced after a low strain (0.1%) to perturb the hydrogel network and followed by another low strain to assess the network’s recovery, the hybrid IPNs hydrogels demonstrated impressive self-healing abilities (Figure 7). The *G*′ of hydrogel discs AF–PHY (Figure 7A,B) returned to approximately 80% of its initial value within seconds after the removal of high strain. Moreover, they can be subjected to several strain cycles, and the storage modulus can completely recover, demonstrating the self-healing ability of the IPN network. These results can be attributed to the dynamic and reversible hydrogen bonding and electrostatic interactions between the AF and PHY polymer networks. Figure 7C shows that the self-healing character of the IPNs is clearly much better than that of the pure PHY polysaccharide hydrogel. In the particular case of BLG-PHY and LYS-PHY, the recovery is even better as time passes, while PHY exhibits a decreasing trend of the recovery with time. APO-PHY hydrogel SEM image (Figure 7D), after self-healing experiments, shows PHY fibers forming a compact structure (background) while brighter protein fibers are practically unchanged.

**Figure 7:**
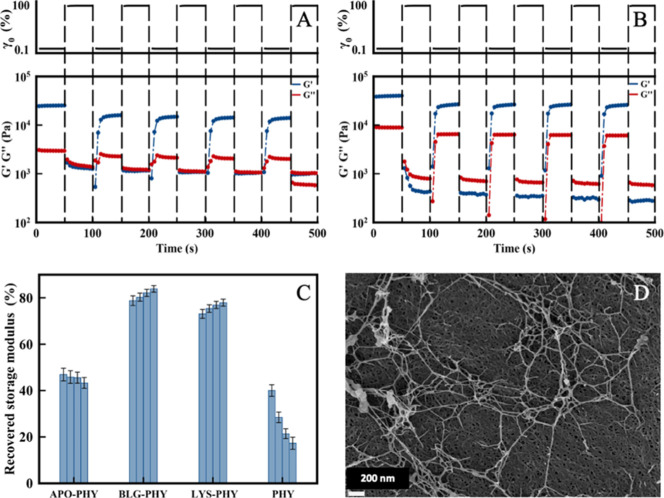
Stepped strain amplitude tests in dynamic oscillatory tests when alternated between γ_0_ = 0.1 and γ0 = 100% over 50 s intervals at *f* = 1.0 Hz and 25 °C. (A,B) Time dependence of the viscoelastic moduli in a five-intervals test for PHY and APO-PHY hydrogels, respectively. (C) Fraction of recovered storage modulus of the hydrogels investigated in this work. (D) SEM image of APO-PHY hydrogel after self-healing experiments.

### 3.7 Real-Time Imaging of Dye-Labeled AF Fibers in AF–PHY Hybrid Hydrogel

A step forward in the development of IPN hydrogels is the ability to control their chemical, physical, and biological performance. To achieve this, it is essential to first investigate their internal microstructures. The most direct route to study these hydrogel microstructures is through characterization methods based on visualization techniques. Thus, together with SEM (cf. Figure 2) to study the allocation of AF protein fibers within the PHY disc, CLSM microscopy was performed. CLSM is a powerful tool for investigating the structure of complex hydrogel systems and offers unparalleled spatial resolution and contrast compared with conventional wide-field fluorescent microscopy.

The appropriate ATTO dyes are fluorophores that are optimally suited to the specific binding of either thiol and amine groups of AF proteins (ATTO maleimide) or carboxy groups in PHY polysaccharides (ATTO amine). This allows for differentiation between PHY and protein nanofibrils. Our group has previously functionalized APO and BLG protein fibers to form 1D fluorescent nanostructures.^62^ In a first experiment, we proceeded to standard functionalization of AF protein, pure PHY, and AF–PHY hybrid hydrogels with ATTO 488 maleimide in order to determine the effective permeation of protein fibers through the PHY disc (Figure 8). The resulting samples were repeatedly washed after functionalization. This synthetic strategy allows us to directly visualize amyloid protein fiber formation and therefore confirm the formation of an IPN network. Figure 8A–C demonstrates that the functionalization of protein fibers with ATTO 488 was successful for pure APO and BLG protein hydrogels (Figure 8A,B). The 3D CLSM reconstruction provided confirmation of homogeneous fluorescent functionalization throughout the material. This was not observed in the case of the pure LYS protein hydrogel, where ATTO488 dye formed aggregates in specific areas (Figure 8C).

**Figure 8:**
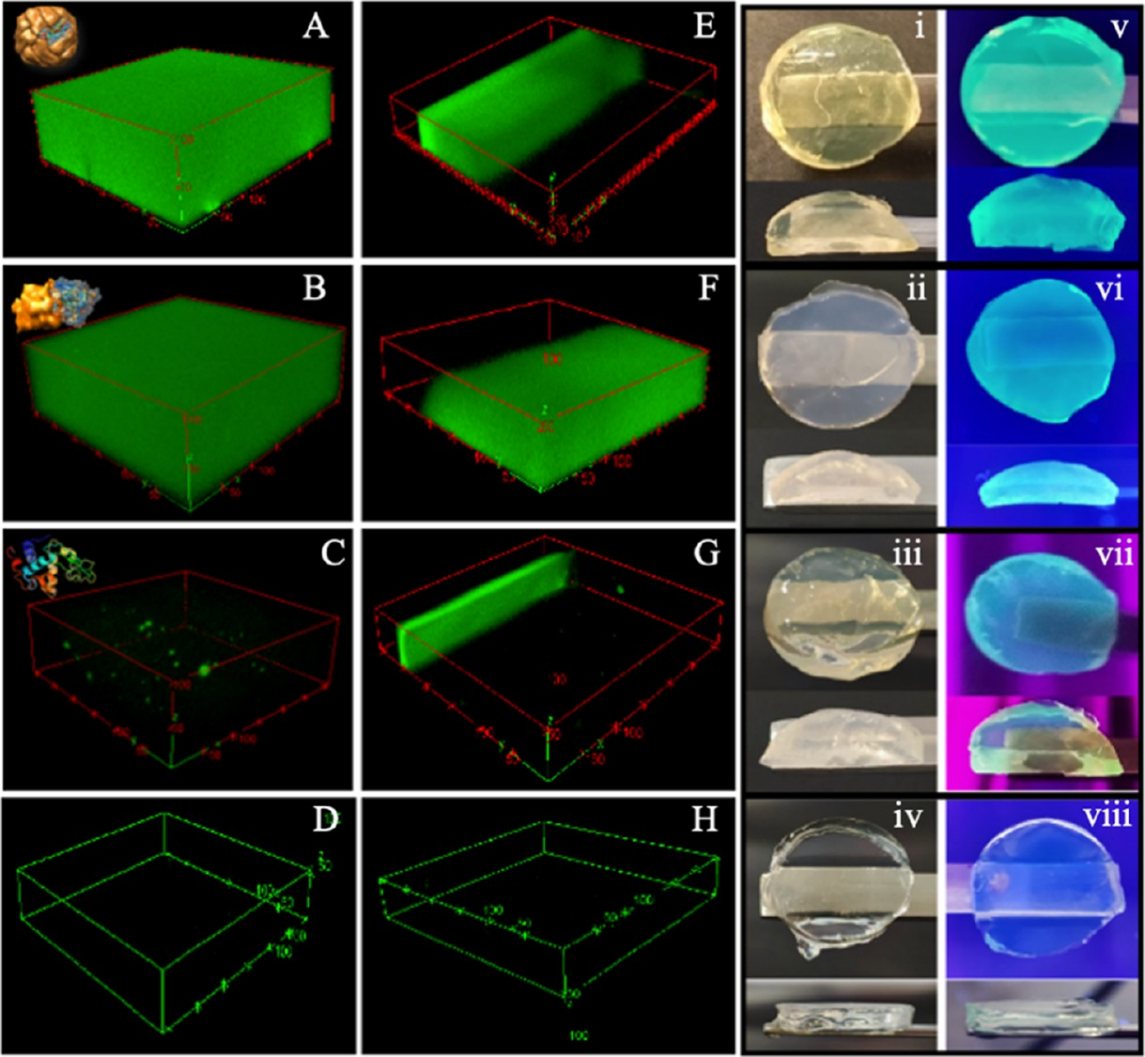
3D CLSM images of (A) ATTO488-APO, (B) ATTO488-BLG, (C) ATTO488-LYS, (D) pure PHY, (E) ATTO488-APO-PHY, (F) ATTO488-BLG-PHY, (G) ATTO488-LYS-PHY, and (H) ATTO488-PHY hydrogels. (i–iv) Hydrogel discs under white light and (v–viii) under UV light irradiation.

For the AF–PHY hybrid hydrogels, the APO-PHY (Figure 8E) and BLG PHY (Figure 8F) hydrogels showed an extremely high density of ATTO488 dye throughout the sample. The side-view images (sum of successive *xy* sections) of the AF–PHY hybrid hydrogels demonstrate that protein fibers have penetrated and invaded the PHY matrix, resulting in the formation of unique fluorescent IPN hydrogels. These hybrid hydrogels showed strong emitted fluorescence. In contrast, in the case of LYS-PHY hydrogel, the fibers only penetrated a few layers of PHY disc, using the same visualization conditions as for (Figure 8G). Figure 8D,H corresponds to pure PHY and ATTO488-PHY, respectively. It can be observed that in the absence of protein fibers, the dye leaches from the PHY network, resulting in an absence of fluorescence. Figure 8(i,v) ATTO488-APO-PHY, Figure 8(ii,vi) ATTO488-BLG-PHY, Figure 8(iii,vii) ATTO488-LYS-PHY, and Figure 8(iv,viii) ATTO488-PHY, discs under white (i–iv) and UV light (v–viii), respectively.

### 3.8 In Situ Imaging of Both Types of Nanofibers in an IPN Hydrogel

We next sought to visualize in situ the two polymeric networks forming the IPN hydrogel (Figure 9). For this purpose, two distinct fluorescent probes that could selectively stain the individual fibers were required. On the basis of their different chemical reactivity, we designed ATTO488 maleimide-tethered amyloid protein fibers (ATTO488-AF) and ATTO647 amine-tethered polysaccharide PHY (ATTO647-PHY). ATTO 647 emits in the red for the polysaccharide network and ATTO 488 emits in the green for the amyloid protein fibers. As can be seen in Figure 9A for the pure PHY hydrogel, ATTO647 can visualize PHY, but the hydrogel was not observed with ATTO488. In contrast, in the APO-PHY hybrid IPN hydrogel, ATTO647 stained PHY fibers, while ATTO488 stained the protein fibers. The orange image is the result of green and red network mixing (Figure 9B).

**Figure 9:**
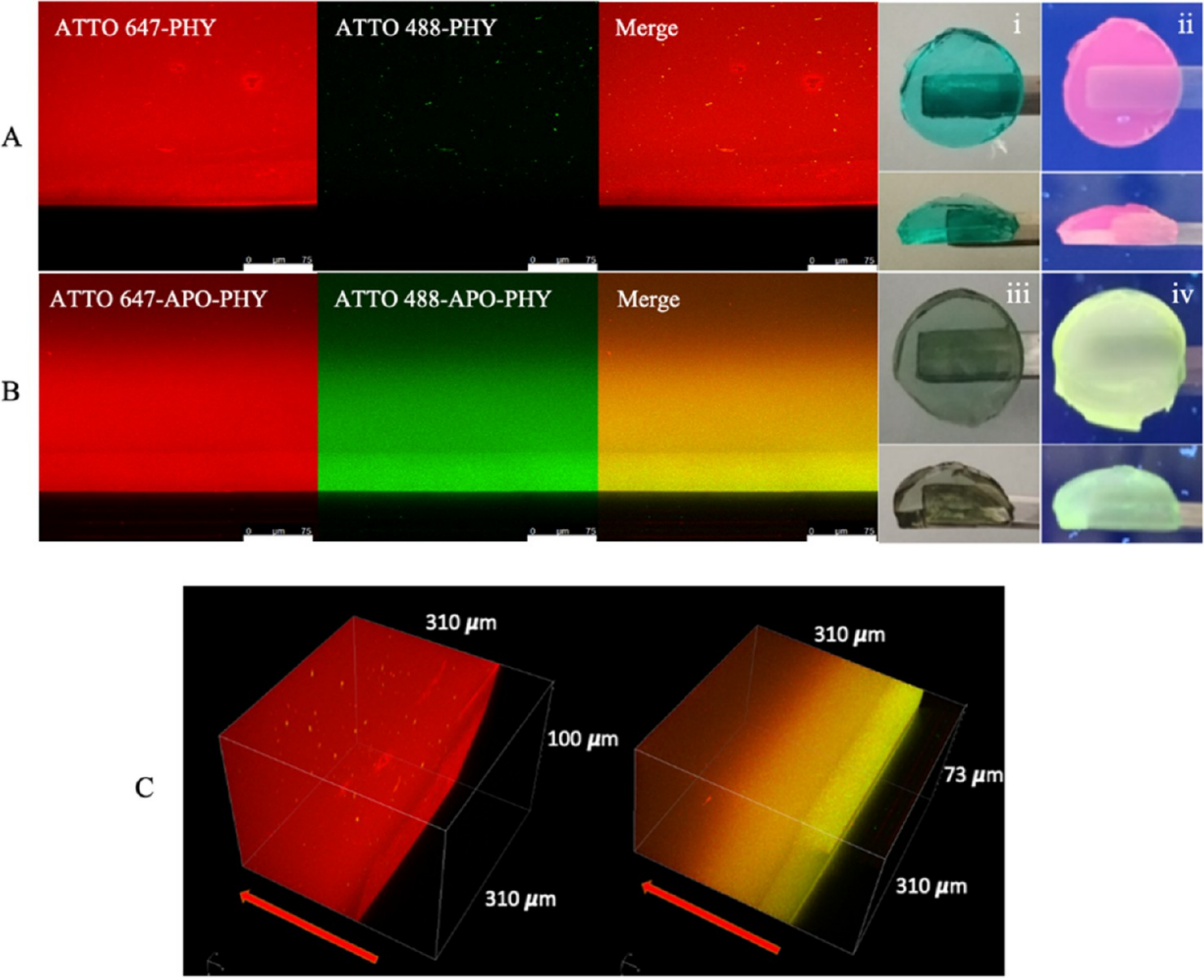
CLSM (A) images of the three-component mixture (PHY/ATTO647/ATTO488). The green and red images were acquired in ATTO647 and ATTO488 channels, respectively. The merged image clearly indicates that PHY pure hydrogel is only functionalized with ATTO647. (B) CLSM images of the four-component mixture (APO/PHY/ATTO647/ATTO488). The green and red images were acquired in ATTO647 and ATTO488 channels, respectively. The merged image clearly indicates that ATTO647-PHY and ATTO488-APO fibers are separately and specifically functionalized with one of the two fluorophores. Scale bars, 75 μm. (C) 3D CLSM image (left) of the three-component (PHY/ATTO647/ATTO488) and (right) of the four-component mixture (APO/PHY/ATTO647ATTO488). The 3D CLSM images are constructed from *z*-stacked *x*–*y* slice images. (i) Three-component and (iii) four-component hydrogel discs under white light and (ii,iv) under UV light irradiation.

In addition to these two-dimensional images, we successfully obtained 3D images (Figure 9C), which clearly visualized the red network in the case of pure PHY hydrogel and orange IPN (merge of green and red networks) in the case of APO-PHY hybrid IPN hydrogel, even in the *z*-axis direction. There is a gradient in orange color, with the intensity of the protein (ATTO 488) being greater on the exterior of the hydrogel disc and that of PHY (ATTO 647) dominating on the interior. Similar results were obtained for the BLG-PHY and LYS-PHY hydrogels (Figure S13).

## 4 Conclusions

The formation of an amyloid-based APO protein hydrogel is reported here for the first time, and its structural and rheological properties are compared to those of BLG and LYS protein hydrogels. The incorporation of APO fibrils introduces a novel strategy for designing protein-based biomaterials. Compared to BLG and LYS, APO fibrils display a broader width distribution (10–160 nm), reflecting greater structural heterogeneity and flexibility that promote the formation of dense, highly interconnected networks. This results in enhanced shear strength and strain-resilient behavior. Despite their density, these hydrogels remain optically transparent, which is a key advantage for biomedical applications such as imaging and cell encapsulation. Protein-based fibrillar systems like these have already demonstrated promise in drug delivery, biosensing, and food structuring due to their ability to form ordered, self-assembled architectures with multifunctional properties.

Additionally, we successfully developed hybrid interpenetrating polymer network (IPN) hydrogels by inducing in situ polymerization of amyloid fibers (AF) within a preformed PHY polysaccharide hydrogel. The incorporation of PHY significantly enhances the structural integrity and mechanical properties of the IPN hydrogels by trapping protein fibers within its network, resulting in a reinforced hydrogel. The diffusion of small protein peptide fragments through the porous PHY network results in the formation of a fibrillar IPN hybrid hydrogel.

Advanced imaging techniques, including HRSEM and DL-based CNN analysis, were employed to differentiate between protein and PHY components within the hydrogel structure. The CNN-generated distribution maps confirmed the presence of AF throughout the hydrogel with a higher concentration at the exterior. Rheological testing demonstrated a synergistic interaction between AF and PHY, yielding enhanced viscoelastic properties that surpass those of the individual components. Moreover, the AF–PHY hydrogels exhibited remarkable self-healing capabilities, with their storage modulus recovering up to 80% within seconds after experiencing high strain.

Three-dimensional CLSM imaging further confirmed the IPN structure of the hydrogels, revealing a higher concentration of protein fibers in the outer regions and a dominance of PHY fibers in the interior. These structural observations were consistent with SEM findings and CNN-based analyses. Additionally, the hybrid hydrogels exhibited a strong fluorescence emission, demonstrating their potential for visualization in biomedical applications.

The novel in situ approach used in this study enables the controlled diffusion and formation of protein fibers within a preformed porous polysaccharide hydrogel. This method presents a promising alternative for fabricating biopolymer-based IPNs with enhanced mechanical performance, outperforming similar materials composed of the same constituents in their native state or produced through simple blending. The resulting AF–PHY hydrogel demonstrates notable mechanical strength (∼1 MPa modulus), optical transparency, and self-healing capabilities, properties that highlight its potential for applications, such as injectable scaffolds, bioimaging platforms, and soft tissue engineering matrices. This method signifies a substantial advancement in the field of hydrogel synthesis, providing a resilient and sustainable approach to the development of high-performance biomaterials that are well-suited for a wide range of biomedical and engineering applications.

## Supplementary Material



## Notes

SEM images, DLS measurements, width fibers determination, rheological properties, ZP measurements, DL images, nitrogen adsorption curves, and CLSM images (PDF)
