# Supplementary material for: Integrating Deep Learning and Real-Time Imaging to Visualize In Situ Self-Assembly of Self-Healing Interpenetrating Polymer Networks Formed by Protein and Polysaccharide Fibers
Source: ACS Appl Mater Interfaces. 2025 Aug 5;17(33):46771–85. doi: 10.1021/acsami.5c11459 (PMC12371690; doi:10.1021/acsami.5c11459)
Supplement: Supplementary file 1 [file am5c11459_si_001.pdf]

Supporting Information

Integrating Deep Learning and Real-Time Imaging to Visualize In Situ Self-Assembly of Self-Healing Interpenetrating Polymer Networks Formed by Protein and Polysaccharide Fibers

Gloria Pelayo-Punzano,[†] Rafael Cuesta,[‖] José J. Calvino,[ǀ] José M. Domínguez-Vera,[†] Miguel López-Haro,[ǀ*] Juan de Vicente,[§] Natividad Gálvez[†*]

[†] Department of Inorganic Chemistry, University of Granada, 18071 Granada, Spain

[‖] Department of Organic and Inorganic Chemistry, EPS Linares, University of Jaén, 23700 Linares, Spain

[ǀ] Department of Material Science and Metallurgy Engineering and Inorganic Chemistry, University of Cádiz, 11510, Cádiz, Spain

[§] Department of Applied Physics, Faculty of Sciences, University of Granada, 18071, Granada, Spain

*Correspondence to: ngalvez@ugr.es, miguel.lopezharo@uca.es

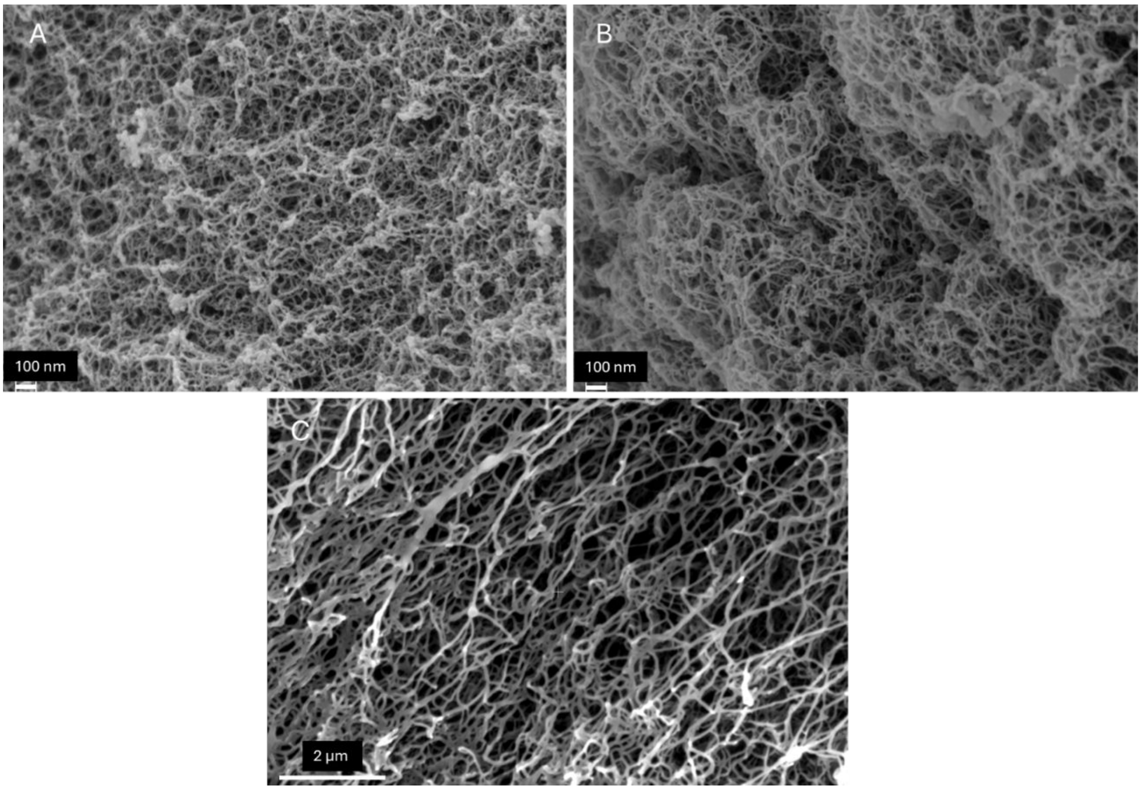

FIGURE S1. A, B, C) SEM images of APO protein at different temperatures, 90°C, 70°C and 50°C, respectively.

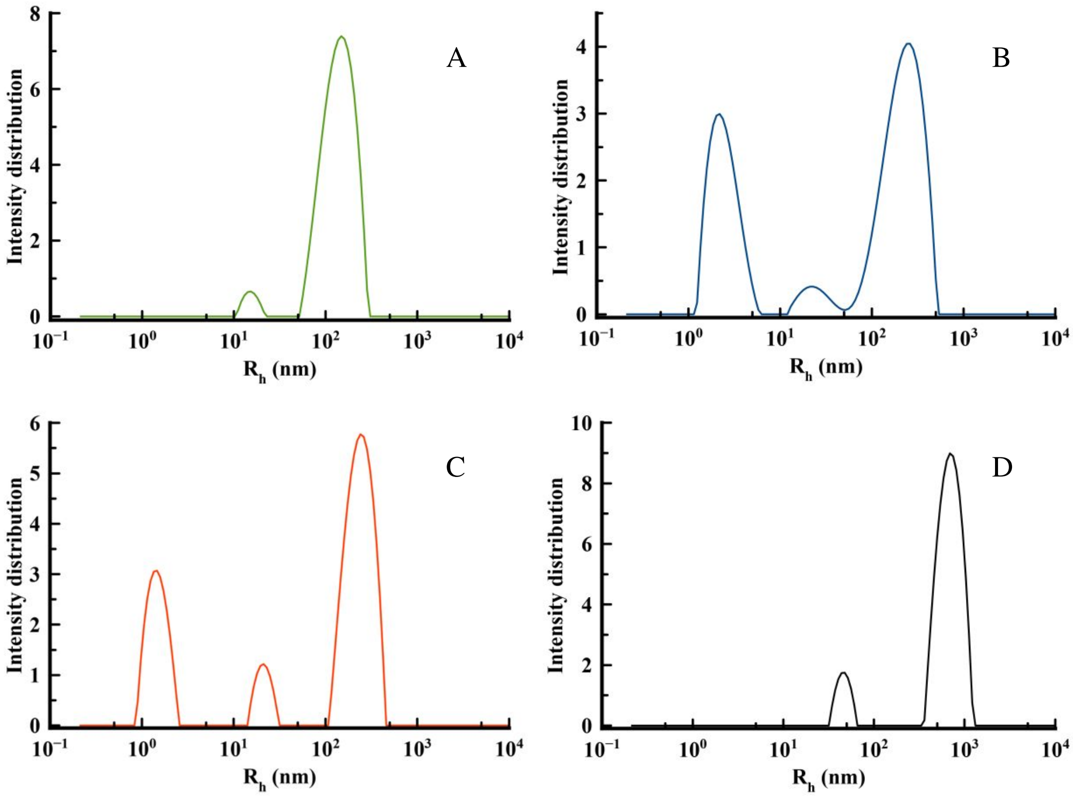

FIGURE S2. A, B, C, D) dynamic light scatering (DLS) measurements of size dispersion of APO, BLG, LYS and PHY pre-gel, respectively.

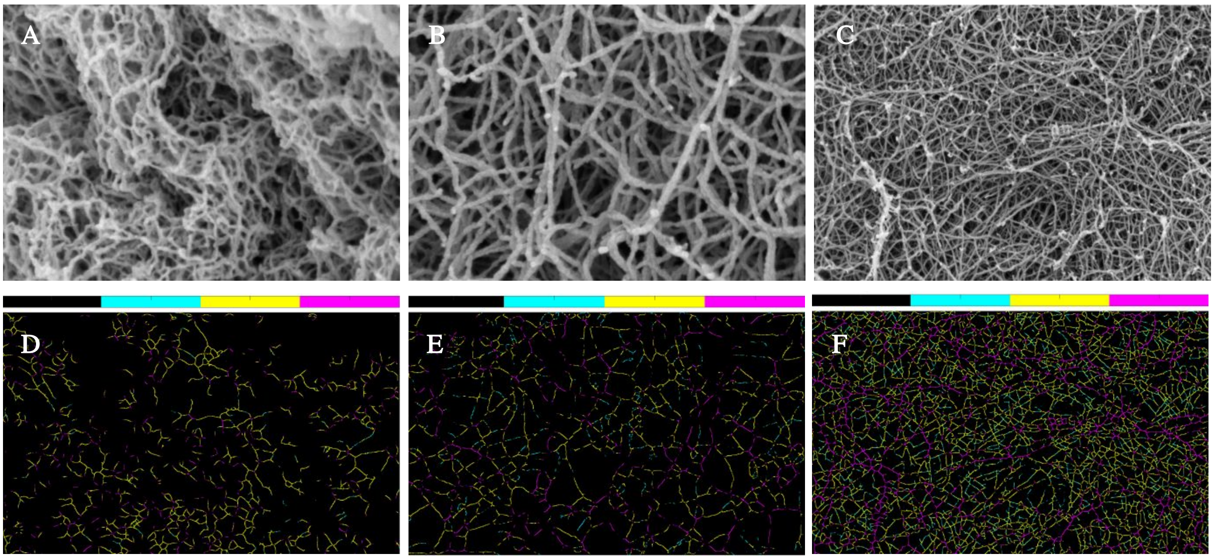

FIGURE S3. A,B,C: HRSEM images of APO, BLG and LYS pure protein hydrogels, respectively. D,E,F, fiber width ranges are represented using a three-color map.

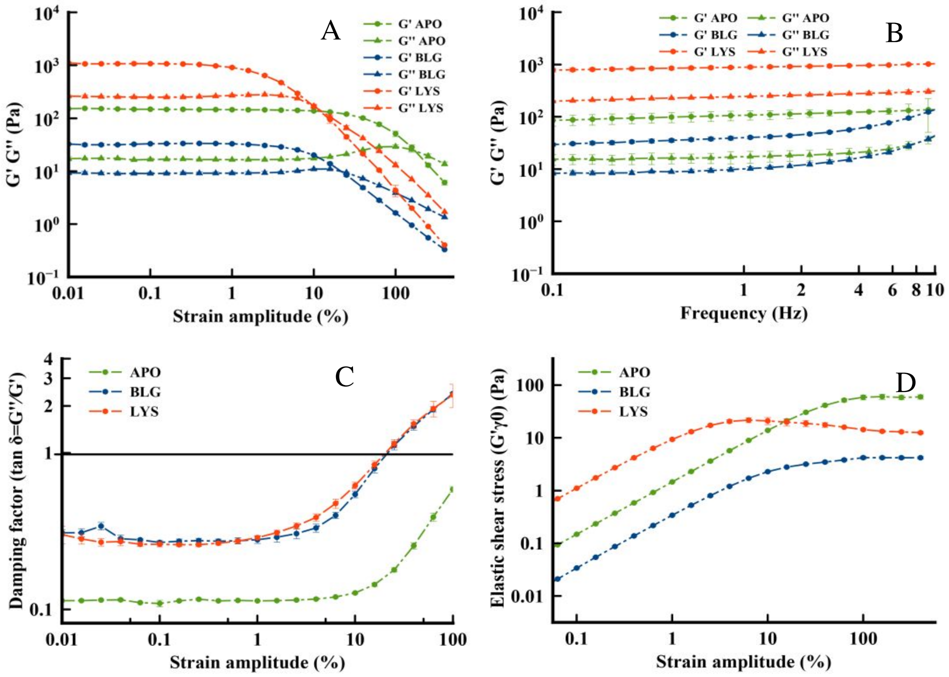

FIGURE S4. Rheological properties of pure protein hydrogels. A) Strain amplitude sweep at a constant frequency (f= 1Hz). B) Excitation frequency sweep at a constant strain amplitude (γ_0= 0.1%). C) Damping factor (tan δ=G''⁄G') as a function of the strain amplitude for the data shown in A). D) Elastic shear stress (G'γ_0) as a function of the strain amplitude for the data shown in A).

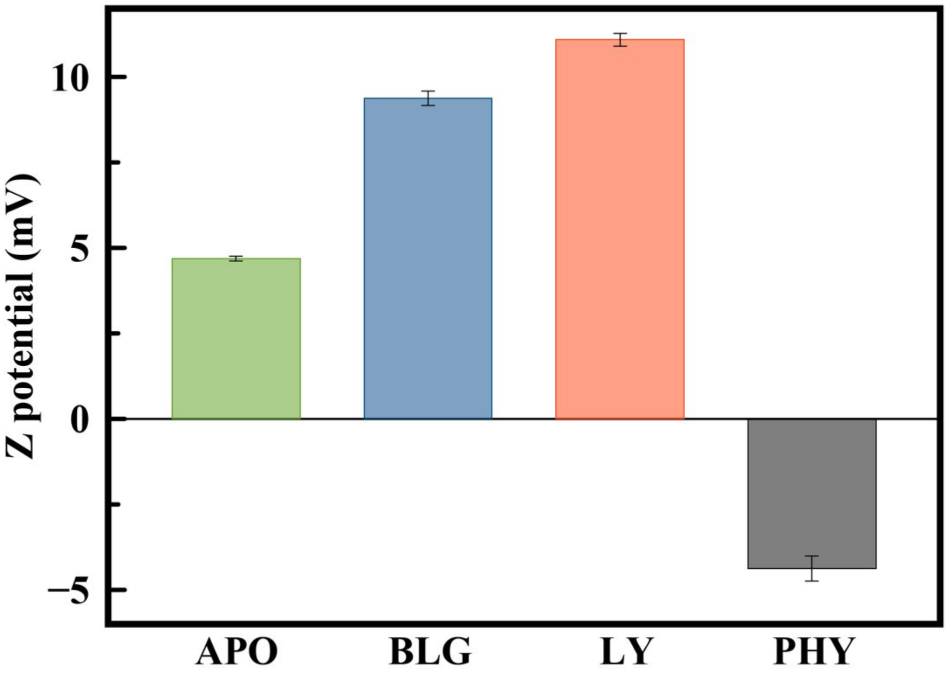

FIGURE S5. Zeta potential measurements of pre-gels.

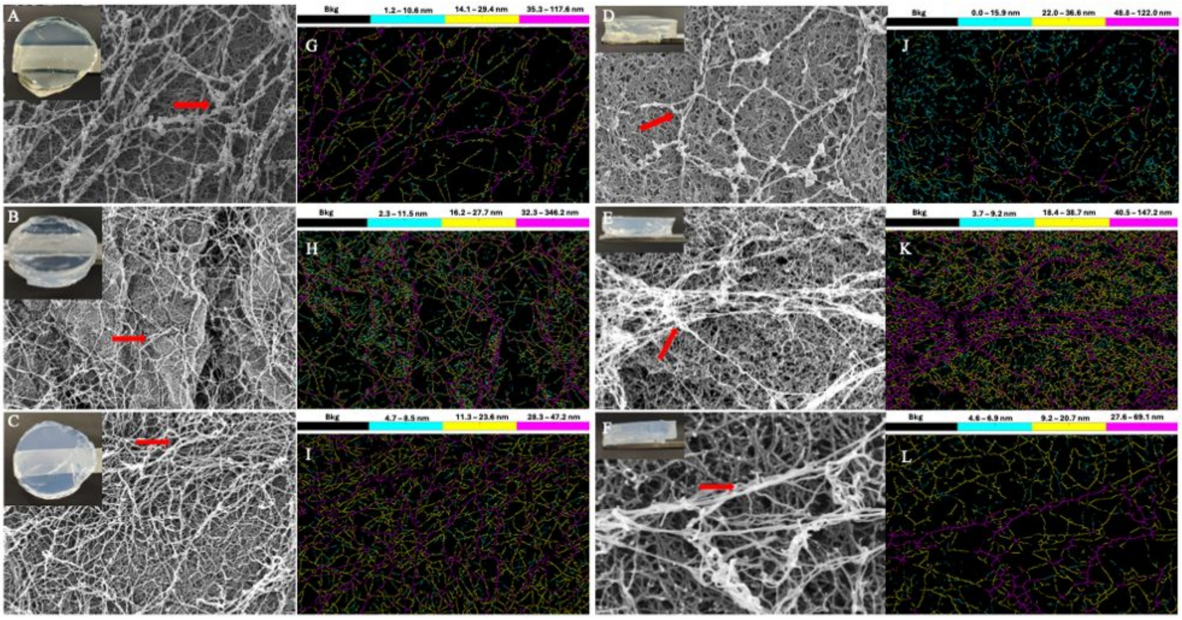

FIGURE S6. A, B, C) disc exterior HRSEM images and D, E, F: disc interior HRSEM images of APO-, BLG, and LYS-PHY IPN hydrogels, respectively. The red arrows highlight the brighter protein fibers. G-L, fiber width ranges are represented using a three-color map.

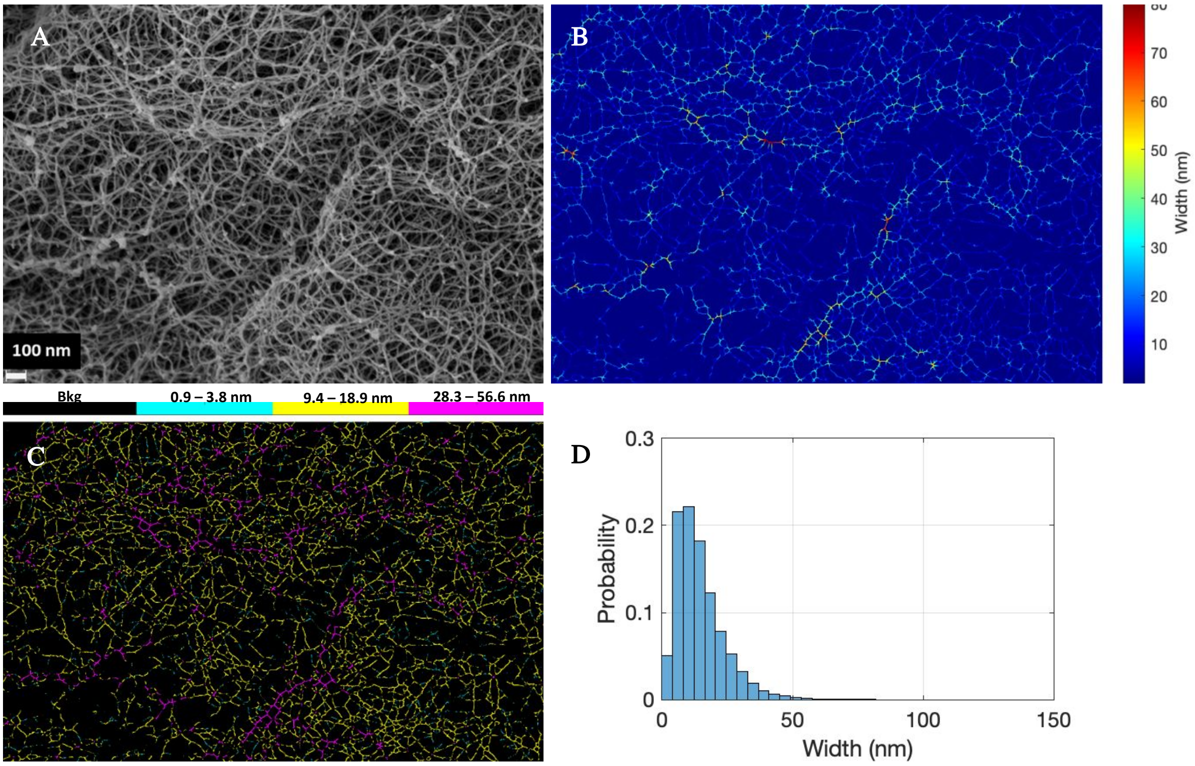

FIGURE S7. A) SEM image of pure PHY hydrogel. B) analysis image of A. C) three-color map image and D) the corresponding width fiber histogram.

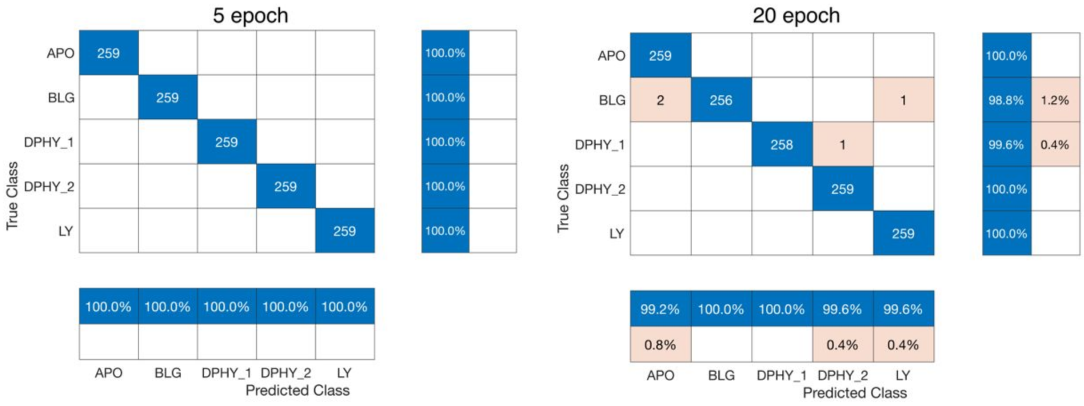

FIGURE S8. Confusion matrix for 5 (left) and 20 epoch (right) obtained from the validation data for the resnet-50 network.

For both matrices, the axes clearly delineate the True Classes and Predicted Classes. The True Class indicates the actual categories to which the samples belong: "APO", "BLG", "DPHY_1", "DPHY_2", and "LY". Conversely, the Predicted Class represents the categories assigned to each sample by the ResNet50 model.

Within the Main Matrix**,** the values denote the count of correctly classified samples. For the 5-epoch model: Each cell along the diagonal consistently displays 259. This indicates that all 259 samples from every class were perfectly identified (e.g., 259 "APO" samples were accurately predicted as "APO"). The absence of any numbers outside the diagonal is particularly significant for this model, as it signifies a complete lack of incorrect predictions; the model did not misclassify any sample across different classes. For the 20-epoch model: While the "APO", "DPHY_2", and "LY" classes still show 259 correct classifications on the diagonal, the "BLG" class shows 256 correct predictions, and "DPHY_1" shows 258. The presence of non-zero off-diagonal values (e.g., 2 "BLG" samples predicted as "APO", 1 "BLG" predicted as "LY", and 1 "DPHY_1" predicted as "DPHY_2") signifies specific instances of misclassification.

The Right Column presents the Recall for each class, computed as *(true positives / (true positives + false negatives))*. For the 5-epoch model: All cells uniformly show 100.0%. This indicates that all samples from every class were perfectly identified, demonstrating the model's flawless ability to recall all instances belonging to each true class. In the case of the 20-epoch model: APO, DPHY_2, LY: These classes maintain a 100.0% Recall, indicating perfect identification. However, BLG shows 98.8% Recall. This is calculated as (256 true positives for BLG / (256 + 2 false negatives (predicted as APO) + 1 false negative (predicted as LY))). The adjacent 1.2% represents the percentage of misclassified BLG simples. Additionally, DPHY_1 shows 99.6% Recall. This is calculated as (258 true positives for DPHY_1 / (258 + 1 false negative (predicted as DPHY_2))). The adjacent 0.4% represents the percentage of misclassified DPHY_1 samples.

Similarly, the Bottom Row illustrates the Precision for each class, calculated as *(true positives / (true positives + false positives))*. For the 5-epoch model: All cells consistently show "100.0%." This confirms that every prediction made by the model was accurate, as there were no false positives. For the 20-epoch model: BLG, DPHY_1: These classes maintain 100.0% Precision, meaning all predictions for these classes were correct. APO: Shows 99.2% Precision. The adjacent 0.8% indicates that when the model predicted "APO", 0.8% of those predictions were actually from other classes (specifically, the 2 "BLG" samples misclassified as "APO"). DPHY_2: Shows 99.6% Precision. The adjacent 0.4% indicates that 0.4% of samples predicted as "DPHY_2" were actually "DPHY_1" (1 "DPHY_1" sample misclassified as "DPHY_2"). **LY:** Shows 99.6% Precision. The adjacent 0.4% indicates that 0.4% of samples predicted as "LY" were actually "BLG" (1 "BLG" sample misclassified as "LY").

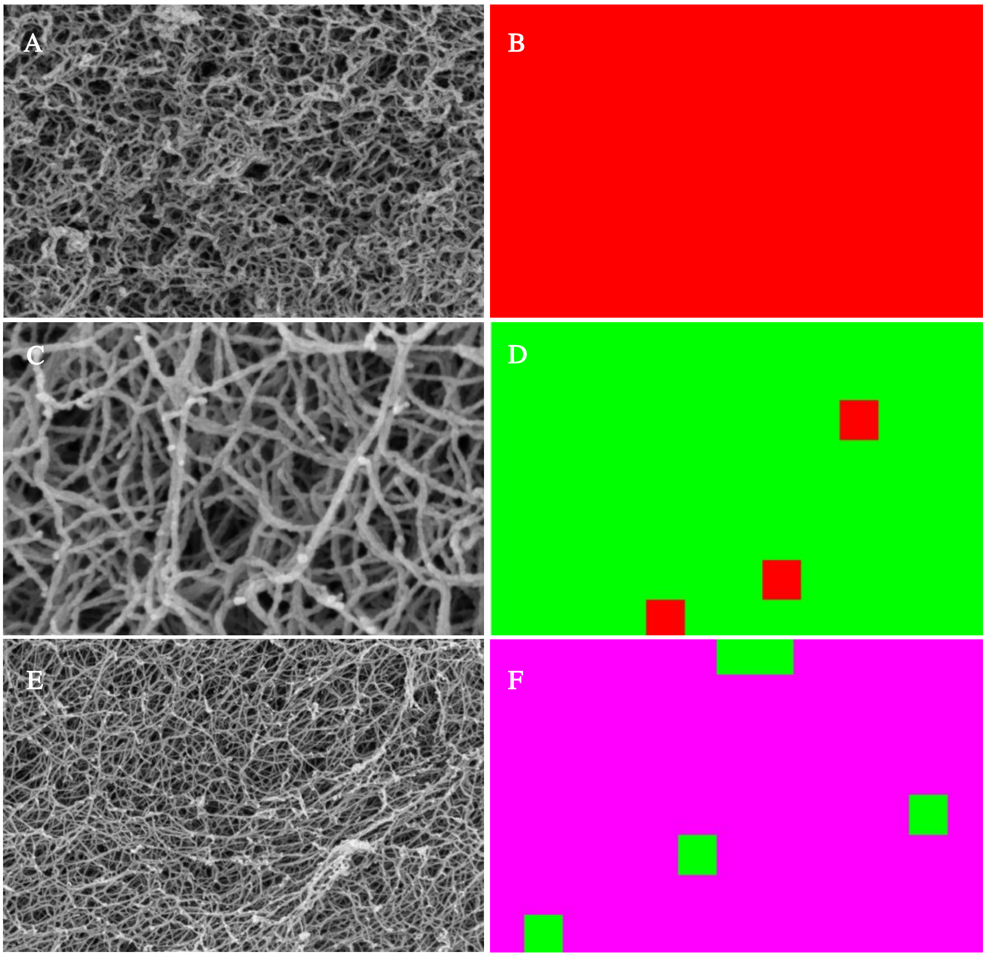

FIGURE S9. A,C,E) SEM images and B,D,F) IA images of APO, BLG, and LYS pure hydrogels, respectively. Red color: APO, green color: BLG, and magenta color: LYS.

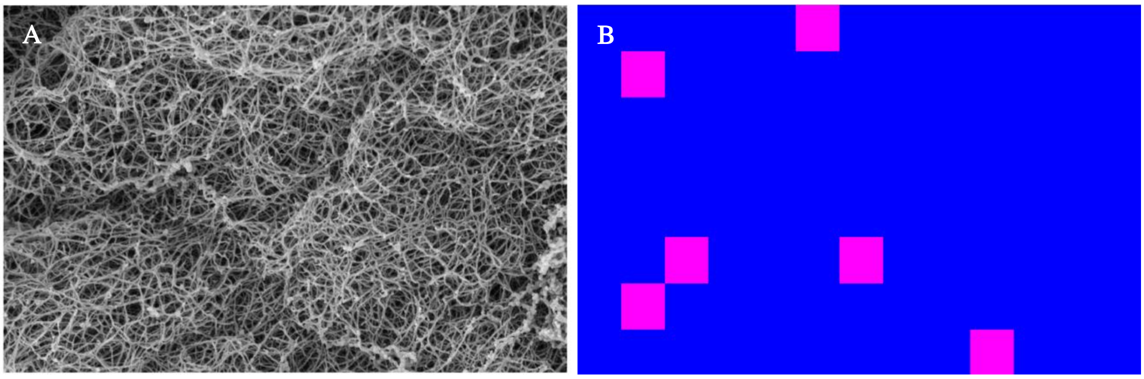

FIGURE S10. A) SEM image and B) IA image of PHY pure hydrogels. Blue color: PHY and magenta color: AF protein.

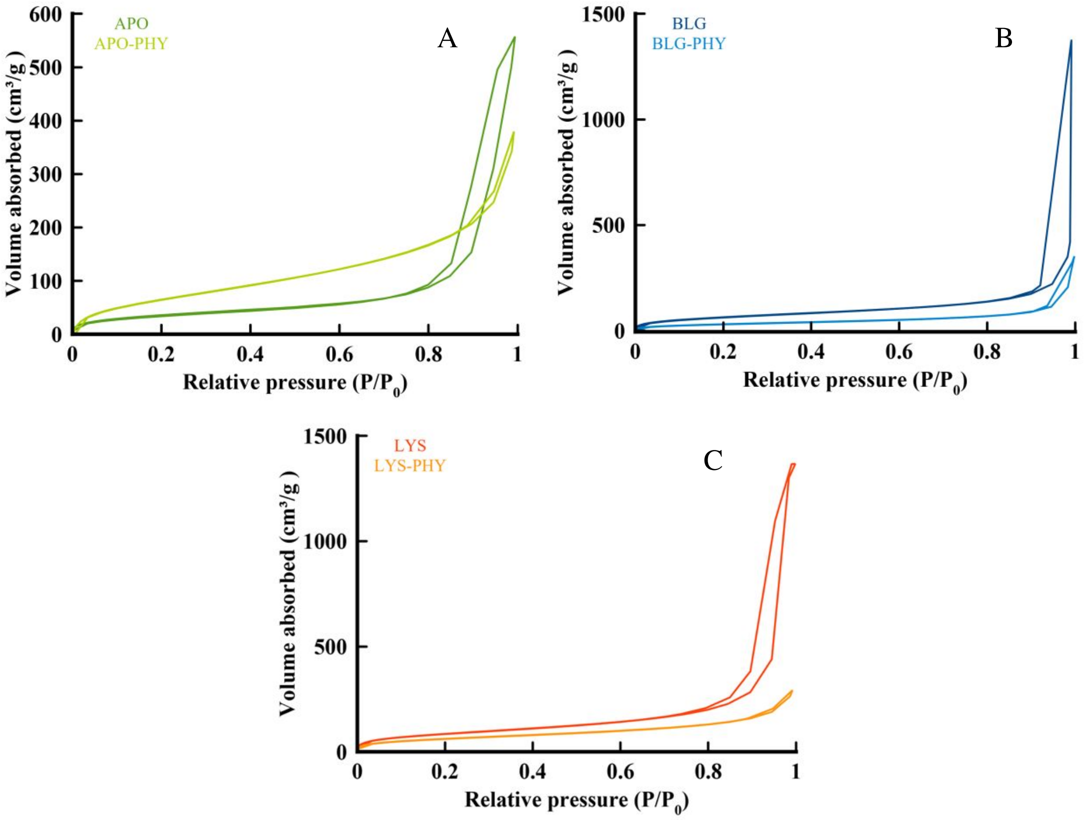

FIGURE S11. Nitrogen gas absorption-desorption isotherms of the AF-PHY hydrogels compared to pure proteins hydrogels: A) APO protein, B) BLG protein and C) LYS protein.

A

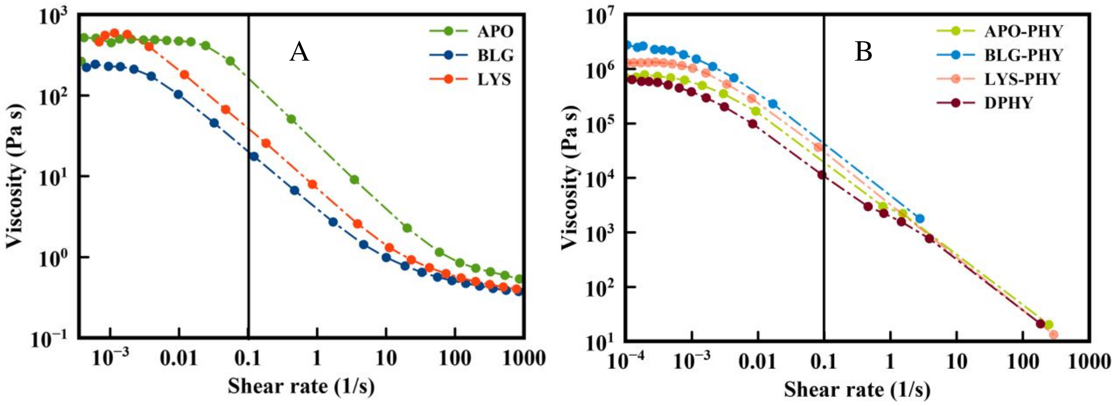

FIGURE S12. Viscosity curves of protein hydrogels (A) and protein-PHY hydrogels (B). Data to the left of the dashed vertical line (1/5 s = 0.2 s^{-1}) are not necessarily in steady state.

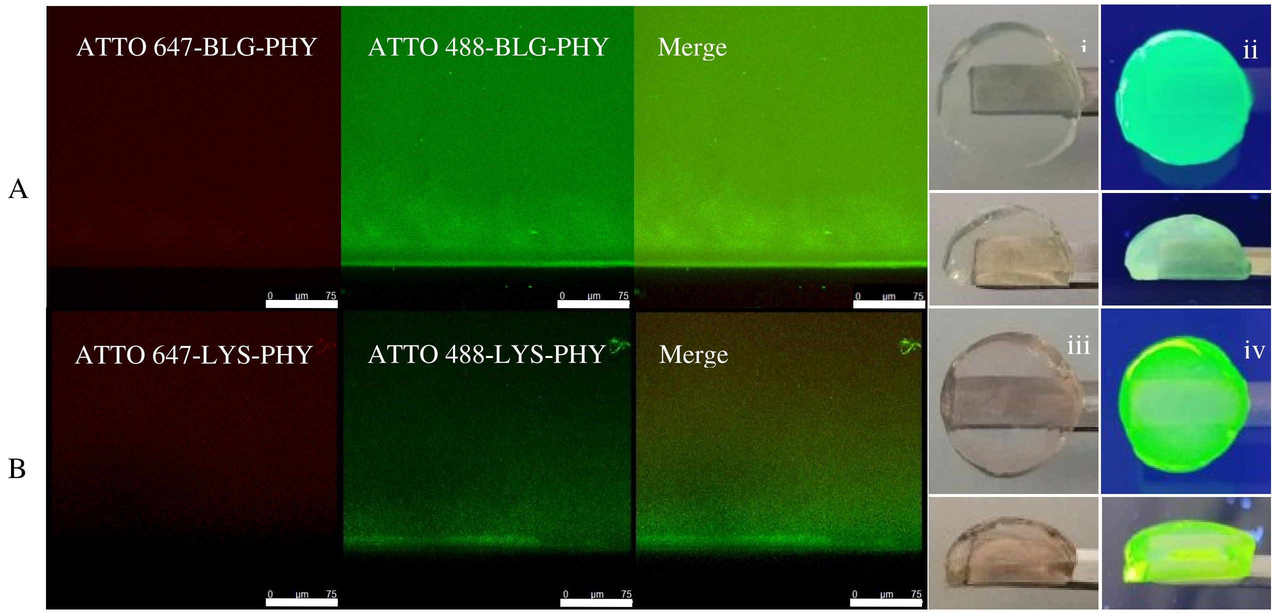

FIGURE S13. CLSM (A) images of the four-component mixture (BLG/PHY/ATTO647/ATTO488). (B) CLSM images of the four-component mixture (LYS/PHY/ATTO647/ATTO488). The green and red images were acquired in ATTO647 and ATTO488 channels, respectively. The merged images clearly indicates that ATTO647-PHY and ATTO488-protein fibers are separately and specifically functionalized with one of the two fluorophores. Scale bars, 75 µm. i) BLG four-component and iii) LYS four-component hydrogel discs under white light and ii, iv) under UV light irradiation.
